# Challenges and Perspectives in Treating Individuals With Musculoskeletal Disorders and Comorbidity: A Systematic Literature Review With a Descriptive Thematic Synthesis

**DOI:** 10.1111/scs.70130

**Published:** 2025-10-03

**Authors:** Laura Celine Rømer, Jacob S. Gandløse, Jane Andreasen, Søren T. Skou, Thorvaldur Skuli Palsson

**Affiliations:** ^1^ Department of Physiotherapy and Occupational Therapy Aalborg University Hospital Aalborg Denmark; ^2^ Aalborg Health and Rehabilitation Center, Aalborg Municipality Aalborg Denmark; ^3^ Public Health and Epidemiology Group, Department of Health Science and Technology, the Faculty of Medicine Aalborg University Aalborg Denmark; ^4^ The Research and Implementation Unit PROgrez, Department of Physiotherapy and Occupational Therapy Næstved‐Slagelse‐Ringsted Hospitals Næstved Region Zealand Denmark; ^5^ Research Unit for Musculoskeletal Function and Physiotherapy, Department of Sports Science and Clinical Biomechanics, Faculty of Health University of Southern Denmark Odense Denmark

**Keywords:** health care, multimorbidity, musculoskeletal disorders, physical therapy, qualitative

## Abstract

**Background:**

The prevalence of comorbidity amongst individuals seeking care for musculoskeletal disorders is rising. This underscores the importance of understanding how health care professionals are managing individuals with musculoskeletal disorders and comorbidity.

**Objectives:**

To analyse the existing literature focusing on healthcare professionals' challenges and perspectives in managing individuals with musculoskeletal disorders and comorbidity.

**Methods:**

A comprehensive search was conducted across four databases: PubMed, CINAHL, Embase and Scopus, focusing on healthcare professionals' experiences of treating individuals with musculoskeletal disorders and comorbidity. The review included studies of a qualitative nature that explored healthcare professionals' experiences in the management of this patient population.

**Results:**

The systematic search yielded 2645 articles, of which five studies were included. All included studies investigated physiotherapists as their target group, while one study also included occupational therapists. Most studies focused on individuals with osteoarthritis and comorbidity. Common challenges identified included a lack of knowledge and training in addressing comorbidities in healthcare professionals, particularly psychological conditions, diabetes and obesity, which was perceived as a barrier for making individualised treatment plans.

**Conclusions:**

This systematic literature review highlights the limited evidence on the experiences of healthcare professionals in treating individuals with musculoskeletal disorders and comorbidity. Findings underscore the need for further training to support clinicians in treating this patient population, and the need for further research exploring the perspectives of healthcare professionals, especially among others than physiotherapists.

## 1 Introduction

The prevalence of people living with two or more long‐term clinical conditions, that is, multimorbidity, is increasing and is estimated to affect more than one in three individuals worldwide [1]. Within this population, musculoskeletal disorders have emerged as a frequently encountered group of conditions [2, 3]. This category encompasses a diverse array of conditions including rheumatological diseases, degenerative conditions, and non‐specific regional pain syndromes [4]. As an example, 62% of people with osteoarthritis treated in primary care have at least one comorbidity, such as hypertension or cardiovascular disease [5].

Healthcare systems are primarily designed to address singular diseases but are likewise ill‐equipped to manage the complexities inherent in addressing multiple concurrent health conditions [1]. This is further supported by clinical guidelines which predominantly concentrate on the management of singular diseases [6, 7], and therefore offer little help to healthcare professionals when providing care for individuals with more than one health condition. Furthermore, clinical trials, which serve as the foundation for evidence‐based practice, frequently exclude individuals with multimorbidity, thereby limiting the applicability of their findings to this population [8]. This systematic exclusion contributes to gaps in knowledge regarding optimal management strategies for individuals with multimorbidity.

However, effective management of multimorbidity likely requires collaboration among multiple healthcare providers [9, 10, 11] as well as a coordinated, person‐centred approach aimed at minimising treatment burden as much as possible [1]. As this is rarely possible, musculoskeletal disorders and multimorbidity have been observed to exert an adverse influence on treatment outcomes [12]. Moreover, individuals with multimorbidity often find that their other health conditions are marginalised during interactions with healthcare personnel [3]. This is aligned with the findings in a recent review, demonstrating that general practitioners experience challenges in employing current clinical guidelines which often focus on individual health conditions and thereby lack guidance in managing individuals with multimorbidity [13]. However, Damarell et al. [13] did not account for individuals with musculoskeletal disorders nor did they include perspectives from healthcare professionals other than general practitioners.

Given that a substantial proportion of individuals living with multimorbidity also have musculoskeletal disorders, it is imperative to gain insight into the experiences of healthcare professionals concerning the treatment of this specific population. The primary objective of this literature review was to conduct descriptive thematic syntheses, focusing on the diverse challenges and experiences of healthcare professionals providing care for individuals with musculoskeletal disorders and comorbidity.

## 2 Methods

This systematic literature review followed the Preferred Reporting Items for Systematic Reviews and Meta‐Analyses (PRISMA) (Appendix S1).

### 2.1 Research Question

This systematic literature review aimed to address the research question: ‘How do healthcare professionals experience the management of individuals with musculoskeletal disorders and comorbidity?’

### 2.2 Search Strategy

The research process commenced with the development of a comprehensive search protocol (Appendix S2) designed to identify relevant literature. The protocol was rooted in the research question and divided into five distinct search blocks: *Musculoskeletal Disorders*, *Multimorbidity/comorbidity*, *Healthcare Professionals* and *Qualitative* search. In the search blocks labelled *Musculoskeletal Disorders* and *Multimorbidity/comorbidity*, synonyms thereof were augmented with pertinent issues associated with these, such as osteoarthritis and diabetes. Search terms were crafted through a combination of controlled subject headings and free‐text exploration utilising resources such as Google Scholar and PubMed. To mitigate the prevalence of extraneous noise in search outcomes, it was stipulated that free‐text terms should appear in either titles or abstracts. Although database‐specific variations in subject headings were acknowledged, the core structure of each search remained consistent, aligning with the established search protocol.

### 2.3 Database Selection

To capture a comprehensive spectrum of literature, the final search was conducted in four key databases: PubMed, CINAHL, Embase and Scopus. The selection of these databases was based on an evaluation of their relevance to the research inquiry, which was conducted collaboratively with a specialised research librarian. The final systematic search was conducted on October 4, 2023, across all the databases (Appendix S4).

### 2.4 Study Selection

To streamline the management of the identified articles, Endnote version 21 (Clarivate, London, UK) was used to identify and remove duplicate entries. The initial screening of studies was based on an evaluation of titles and abstracts guided by predefined inclusion criteria. Subsequently, a thorough examination of full‐text articles was conducted. Two investigators (L.C.R. and J.S.G.) independently performed the screening process according to the predetermined criteria. Articles deemed relevant based on title and abstract screening were uploaded to a web‐based platform for systematic literature reviews and meta‐analysis, Rayyan (www.rayyan.ai). This allowed the two researchers to identify inconsistencies after a blinded screening process. In cases of discrepancies, the two researchers discussed their findings to reach consensus. Any potential disagreements were resolved by the third researcher (T.S.P). To ensure the inclusion of all potentially relevant studies not identified through the systematic literature search, a chain‐search technique was employed. This technique involved screening the reference lists of the selected studies based on the predefined selection criteria. Studies meeting the criteria for full‐text review were subsequently incorporated. Additionally, a citation search of the studies included in the systematic search was conducted to identify any relevant studies not captured in the initial search.

### 2.5 Inclusion Criteria

For inclusion, the study populations had to consist of healthcare professionals working with patients with musculoskeletal disorders in conjunction with the presence of comorbidity. The included studies had to be of qualitative nature, employing interviews, field observations, and analysis of textual data as their primary research methods if they investigated healthcare professionals' experiences on treatment. All interview formats were included (e.g., one‐on‐one, face‐to‐face, virtual and focus group interviews). Publications in English, German, Norwegian, Swedish and Danish languages were included.

### 2.6 Thematic Synthesis

The included studies were summarised in tables, providing information on the country, population/theme, sample size, study design and purpose. A more detailed table was also included, outlining the study design, aim, population, methods, results and conclusions. A descriptive thematic synthesis approach, as outlined by Thomas and Harden [14], was then applied to identify and analyse themes related to the research question. Themes were first individually identified within each study, followed by a systematic process of recognising similarities across studies, allowing for an integrated synthesis of the results. The findings are presented collectively and supported by quotations from the qualitative analyses in the included articles. Furthermore, all statements from the included studies were extracted and compiled in a Appendix S6.

### 2.7 Quality Assessment and Synthesis of Results

Included articles were assessed for quality by the principal investigator (L.C.R.) utilising the Critical Appraisal Skills Programme (CASP) framework for qualitative research [15]. CASP was selected for its widely recognised applicability in qualitative research and its systematic evaluation of key aspects such as study design, data collection and analysis, ensuring a rigorous and structured quality assessment of the included studies [16].

## 3 Results

The systematic search yielded 2645 results, after removing duplicates. Of these, 38 were identified as relevant based on title and abstract screening and subsequently proceeded to full‐text examination. After a thorough review of the full‐text articles, five studies were ultimately included in the review (Table 1).

**TABLE 1:** List of included studies containing the study's country, population/theme, design and purpose.

Reference/number of informants (*n*)	Country	Population/Theme	Type of publication/design	Purpose
Gibbs et al. [17] *n* = 19	Australia	Physiotherapists from specialised osteoarthritis services	Qualitative/individual interviews	To investigate physiotherapist perspectives of knee‐ and hip osteoarthritis care.
Hemmings and Soundy [18] *n* = 8	United Kingdom	Physiotherapists working with individuals with severe mental illness and comorbid physical health problems such as back pain	Qualitative/individual and focus group interviews	To explore experiences of physiotherapists in providing care for those with both physical and mental health complaints to identify barriers and facilitators to care.
King et al. [19] *n* = 18	Canada	Physiotherapist and occupational therapists working with patients with knee osteoarthritis	Qualitative/individual interviews	To understand the extent to which arthritis therapists consider type 2 diabetes mellitus (T2DM) when treating persons with knee osteoarthritis and comorbid T2DM, and barriers to doing so.
Lawford et al. [20] *n* = 7	Australia	Physiotherapists working with patients with knee osteoarthritis and comorbid obesity	Qualitative/individual interviews	To explore challenges among physiotherapists associated with implementing a home‐based strengthening exercise programme for individuals with knee osteoarthritis and comorbid obesity.
Teo et al. [21] *n* = 22	Australia	Physiotherapists working with knee osteoarthritis	Qualitative/individual interviews	To investigate physiotherapist perspectives of delivering care for people with knee osteoarthritis.

**FIGURE 1:**
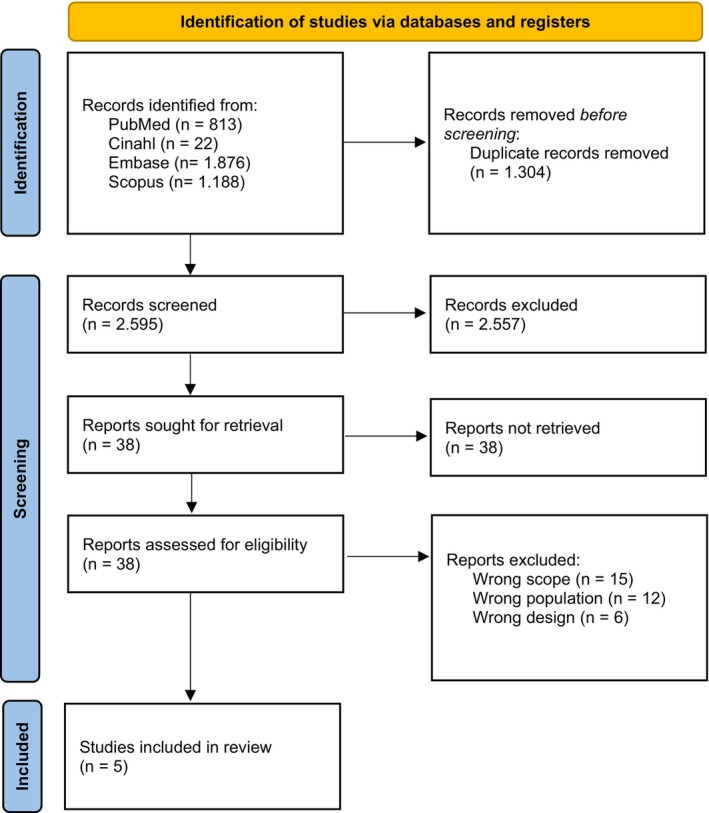
Flowchart demonstrating the findings in each step of the screening process.

Following the full‐text screening process, the two investigators agreed on 82%. The discrepancies were resolved involving the third investigator. A comprehensive overview of the selection process is outlined in Figure 1. No studies were included using the chain‐search technique, including the identification of references and citation searches. A detailed overview of the included studies, including their objectives, methodologies, results and conclusions, can be found in Appendix S3. For an overview of the 33 excluded studies and reasons for exclusion, see Appendix S5.

### 3.1 Characteristics of Included Studies

All included studies used interviews to explore the experiences of healthcare professionals in the context of treating individuals with musculoskeletal disorders and comorbidity [17, 18, 19, 20, 21]. All included studies included physiotherapists as their target group, where one study included occupational therapists [19]. Four of the studies specifically focused on the treatment of osteoarthritis [17, 19, 20, 21], and one study investigated the treatment of individuals with other physical disorders, including chronic back pain [18]. The main comorbidities in the studies were obesity and diabetes [17, 19, 20, 21]. Four of the studies were rated as high quality, while one study was assessed as having fair quality (Table 2).

**TABLE 2:** Quality assessment of included studies—CASP checklist.

	Gibbs et al. [17]			Hemmings and Soundy [18]			King et al. [19]			Lawford et al. [20]			Teo et al. [21]		
	Yes	No	Can't tell	Yes	No	Can't tell	Yes	No	Can't tell	Yes	No	Can't tell	Yes	No	Can't tell
Was there a clear statement of the aims of the research?	X			X			X			X			X		
Is a qualitative methodology appropriate?	X			X			X			X			X		
Was the research design appropriate to address the aims of the research?	X			X			X			X			X		
Was the recruitment strategy appropriate to the aims of the research?	X			X			X			X			X		
Was the data collected in a way that addressed the research issue?	X			X			X			X			X		
Has the relationship between researcher and participants been adequately considered?	X			X			X			X			X		
Have ethical issues been taken into consideration?	X			X			X			X			X		
Was the data analysis sufficiently rigorous?	X				X		X			X			X		
Is there a clear statement of findings?	X				X		X			X			X		
How valuable is the research?	The authors discuss the study's contributions in relation to existing knowledge, contextualise the results within current practices and relevant literature, and identify new areas where further research is needed. The transferability to other contexts is also considered.			The authors discuss the study's contributions in relation to existing knowledge, contextualise the results within current practices and relevant literature, and identify new areas where further research is needed. The transferability to other contexts is also considered.			The authors discuss the study's contributions in relation to existing knowledge, contextualise the results within current practices and relevant literature, and identify new areas where further research is needed. The transferability to other contexts is also considered.			The authors discuss the study's contributions in relation to existing knowledge, contextualise the results within current practices and relevant literature, and identify new areas where further research is needed. The transferability to other contexts is not considered.			The authors discuss the study's contributions in relation to existing knowledge, contextualise the results within current practices and relevant literature, and identify new areas where further research is needed. The transferability to other contexts is also considered.		
Quality	High			Fair			High			High			High		

### 3.2 Synthesis of Findings

#### 3.2.1 Theme 1: Knowledge and Training Deficits and Educational Needs

Healthcare professionals frequently encounter challenges when treating individuals with musculoskeletal disorders and comorbidities [17, 18, 19, 20, 21]. One consistently emerging barrier is the perceived lack of knowledge and training among healthcare professionals in addressing these comorbidities [18, 19]. For instance, they often feel ill‐equipped to handle conditions such as mental disorders and diabetes, as one professional expressed, ‘I can't go open that can of worms because I don't know what to do with it’ [19]. This deficiency in expertise can hinder their ability to provide comprehensive care and support for patients dealing with concurrent health issues [18, 19].

Physiotherapists consistently express a significant relationship between patients' comorbidities and their ability to fully commit to osteoarthritis exercise regimens [17, 20, 21]. Among these comorbidities, obesity seems to be one of the most challenging for physiotherapists [17, 20, 21]. Obesity significantly hinders an individual's capacity to engage in recommended exercises [20, 21] and thereby presents a complex task for physiotherapists to adapt treatment programmes to accommodate these challenges [20].

Recognising the knowledge gap, some physio‐ and occupational therapists express a desire for further education, acknowledging that it would enhance their ability to navigate the complexities of concurrent health issues [19]. They emphasise the importance of gaining a better understanding of how conditions like diabetes are managed, the medications involved, and the impact of exercise on such conditions [19]. This desire for further education is driven by the aspiration to provide more comprehensive care and support to patients dealing with musculoskeletal disorders and comorbidities, as one therapist noted, ‘I would definitely feel more comfortable having more education around how diabetes in general is managed and the different medications’ [19].

#### 3.2.2 Theme 2: Capacity and Motivation Challenges

Healthcare professionals struggle with a lack of capability and motivation when it comes to addressing comorbidities [18, 19]. They highlighted structural limitations in the healthcare system and how limited resources make it difficult to provide adequate treatment, *‘So, the chronic disease management plans – you only get five sessions for allied health interventions, and so when you've got other comorbidities… five probably doesn't quite cut it for the year when a lot of people don't have the money to go privately’* [17]. This challenge can result in difficulties in fully committing to the treatment of patients with musculoskeletal disorders and concurrent conditions [18, 19]. For example, some physio‐ and occupational therapists find it challenging to provide guidance on the optimal coordination and integration of osteoarthritis treatment with concurrent diabetes management, as they feel ill‐prepared for this aspect of care [19]. This lack of capability and motivation can result in certain comorbidities being ignored, and healthcare professionals may be uncertain about how to incorporate them into the treatment plan [18, 19]. As one therapist described, ‘I think I currently just ignore that piece [comorbidity]. If they tell me they've got diabetes, I just write it in their medical history part, and I don't address it, or have it influence any of my recommendations, really’ [19].

#### 3.2.3 Theme 3: Scope of Practice Considerations

In the treatment of individuals with multimorbidity, some healthcare professionals do not consider the management of certain comorbid conditions as part of their professional role [19, 21]. This results in a selective focus on specific health issues while potentially neglecting others [19, 21]. For example, some physio‐ and occupational therapists may exclude the management of diabetes from their responsibilities when treating patients with osteoarthritis: ‘I don't think that it would be my role [to optimise concomitant diabetes in patients with OA]’ [19]. Additionally, some physiotherapists consider the management of obesity to fall outside their professional scope and would rather refer patients to nutrition experts, such as dietitians: ‘I think that there are other health professionals who have a better way of approaching it compared to me’ [21]. The same approach is additionally applied to medication counselling, where healthcare professionals often choose to refer patients to other specialists in cases involving medication management [21]. This selective approach raises questions about how it may negatively impact patient care and outcomes.

#### 3.2.4 Theme 4: Emotional Impact and Concerns

The challenges and uncertainties that healthcare professionals face when addressing comorbidities can lead to concerns about the potential consequences for their patients. The uncertainty about how to manage certain comorbid conditions, such as obesity, can evoke feelings of inadequacy and guilt among professionals [17, 21]. For instance, physiotherapists may feel that their lack of skills in discussing obesity management with patients affects their ability to provide effective care [19, 21]. This is for example, reflected in ‘*If I understood that a bit better, then I might be able to communicate that [sic importance of weight management] to them better’* [19]. The challenges of managing obesity and other health issues can lead to feelings of helplessness among healthcare professionals, as they struggle to identify effective strategies and may opt to focus on education rather than specific guidance on managing these conditions [21].

## 4 Discussion

The review included five qualitative studies that employed in‐depth interviews to explore the experiences of healthcare professionals regarding the treatment of individuals with musculoskeletal disorders and comorbidity. The contributions of the included studies showed resemblance and were found to be complementary rather than conflicting in relation to challenges and perspectives. Despite an understanding of the negative association between multimorbidity and treatment outcomes [12], the synthesis derived from the studies highlights challenges in different areas, that is, knowledge, skills, attitudes and insecurity in their practices; factors that all may negatively affect a comprehensive treatment of these patients. Research shows that patients with multimorbidity experience several burdens directly related to the approach from their healthcare provider [22], underlining the importance of addressing the challenges in clinical practice.

### 4.1 Health Care Professionals Lack Competencies in Addressing Comorbidity in Musculoskeletal Disorder

The overarching barrier in treating individuals with musculoskeletal disorders and concurrent health conditions lies in the perceived lack of competencies and knowledge regarding comorbidities among healthcare professionals [17, 18, 19, 20, 21]. This deficiency complicates the ability of healthcare professionals to design individualised treatment plans that encompass all the patients' health issues [17, 19, 20]. This challenge is further exacerbated by the exclusion of individuals with multimorbidity from many clinical trials, limiting the available evidence to guide healthcare professionals in managing these complex cases [8]. These findings are corroborated by a recent clinical trial aimed at evaluating the feasibility of exercise therapy and self‐management among individuals with multimorbidity [1], as well as by a qualitative study that explored perspectives on self‐management and exercise behaviour in the context of multimorbidity [23]. The studies highlighted the urgent need for enhanced training in managing multiple health conditions, with a particular emphasis on the role of physiotherapists [1]. Simultaneously, healthcare professionals encounter challenges in facilitating behaviour change in individuals with multimorbidity due to limited resources, including a deficiency in knowledge about managing multimorbidity [23].

The current findings demonstrated a perceived inability to manage psychological conditions [18, 19, 24]. This is corroborated by previous systematic reviews investigating the knowledge, behaviours, attitudes and beliefs of physiotherapists concerning the use of psychological interventions in their practice [24, 25]. The reviews demonstrated that physiotherapists managing musculoskeletal pain encounter time constraints, lack of training and lack of confidence in utilising relevant psychological methods for patients with unhelpful thoughts or emotional distress. In some cases, this may lead to a stigmatisation where the patient's lack of compliance with treatment is considered the reason for the lack of effect [24]. Consequently, it may be worth considering whether physiotherapists should receive training to address certain psychological conditions to effectively incorporate them into the treatment of musculoskeletal disorders. Further, the presence of multimorbidity significantly influences clinical decision‐making, as guidelines and treatment recommendations often fail to account for the complexity of coexisting conditions. Current clinical guidelines primarily focus on single diseases, making them difficult to apply in multimorbid patients [6, 26]. This misalignment between guidelines and real‐world patient presentations may contribute to healthcare professionals' uncertainty in managing patients with both musculoskeletal disorders and additional chronic conditions.

Individuals with obesity perceive various barriers to exercise, such as competing health problems and a lack of enjoyment and motivation [27]. Additionally, obese individuals often feel insecure and uncomfortable engaging in physical activity [28] and tend to lack trust in their therapist and feel stigmatised [29]. This is somewhat understandable but likewise unfortunate, considering that many clinicians managing musculoskeletal disorders face challenges amongst people with obesity [17, 20, 21]. Given that musculoskeletal disorders, particularly osteoarthritis, are highly prevalent in individuals with multimorbidity [4], a more integrative treatment approach is warranted. Research suggests that 62% of individuals with osteoarthritis have at least one additional chronic condition, such as hypertension or heart disease [5], further underscoring the need for a holistic management strategy that acknowledges the broader health profile of the patient rather than focusing solely on musculoskeletal pain. Considering that osteoarthritis has the highest occurrence of comorbidities [30], where 62% have at least one comorbidity (e.g., hypertension or heart disease) [5] and the strong association between painful osteoarthritis and obesity [31] underscores that a successful outcome likely requires a broad approach where clinicians need to integrate several elements into the management plans. Educational efforts and clinical training, focusing on the importance of the presence of comorbidity in musculoskeletal disorders, are clearly warranted to accommodate the needs of clinicians, as described above.

### 4.2 Management Strategies in Musculoskeletal Disorders Need to Have a Broad Approach

Contemporary guidelines highlight the importance of adopting a patient‐centred approach in managing musculoskeletal disorders [32]. Inevitably, this requires a need to address all the patients' health issues, even though the referral only indicates a single condition. A specialisation within the healthcare system has clear benefits but at the same time may fall short when the patient has other, parallel health conditions. This is evident by the lack of confidence healthcare providers have in addressing other diseases as identified in the current study. This systematic literature review sheds light on the challenges encountered in their clinical practice. These barriers underscore the need for enhanced training and competencies to effectively address comorbidities in the context of musculoskeletal disorders. Further research is essential to better reflect the patient population and support physiotherapists in delivering holistic care. Exercise and physical activity are well‐documented as effective, safe interventions for individuals with multimorbidity [33, 34]. Given their expertise in integrating physical activity, physiotherapists play a key role in managing musculoskeletal pain while addressing coexisting conditions. By tailoring exercise programmes to multiple conditions, they may help reduce treatment burden, improve care coordination and enhance health outcomes [35]. Future research should explore how such approaches can be tested and refined through implementation research, including trials of interdisciplinary interventions aimed at improving integration of care for people with comorbidities. In addition, mixed‐methods and quantitative studies could help assess the effectiveness, feasibility and contextual factors influencing such interventions in real‐world clinical settings.

## 5 Methodological Considerations and Limitations

A key consideration regarding the trustworthiness of the synthesis lies in the process of thematic synthesis. One limitation is the inherent subjectivity involved in interpreting and organising the themes, as the method relies on the researchers' judgement, which may introduce bias. Additionally, the heterogeneity of the studies (Table 1 and Appendix S3) could have shaped the themes that emerged and may result in certain studies exerting a disproportionate influence on the identified themes. This raises the concern that some themes may be driven more by specific studies rather than reflecting patterns that consistently emerge across the body of literature. Despite these limitations, the use of descriptive thematic synthesis provided a structured framework for organising the findings, which allowed for meaningful conclusions to be drawn across the studies. The included studies only reported experiences among physiotherapists and occupational therapists. It is therefore not possible to determine whether other health professions, for example, physicians, nurses and psychologists, experience similar challenges in managing people with musculoskeletal disorders and comorbidity. The transferability of the current results to a broader spectrum of health care professionals should therefore be done with caution. However, the themes identified are likely to be relevant across disciplines involved in the care of individuals with complex health needs [11].

Most of the included studies focused on osteoarthritis and only a limited range of comorbidities. Therefore, this review may not encompass the full diversity of musculoskeletal disorders, nor comorbidities where different conditions may present unique challenges, requiring varying treatment approaches. As such, the insights gained from this review may not be universally applicable to all musculoskeletal disorders. In addition, the small number of included studies (*n* = 5) limits the richness and diversity of the findings, which may further constrain the broader applicability of the results.

Finally, none of the included studies investigated the experiences of healthcare professionals managing patients with multiple distinct chronic illnesses simultaneously but rather focused on a single comorbidity alongside the musculoskeletal disorder. The findings, however, provide an overview of the current research in the field and point to important and informing themes that should be addressed further in future research.

## 6 Conclusion

This systematic literature review highlights the experiences and distinct challenges that healthcare professionals face in managing patients with musculoskeletal disorders and concurrent multimorbidity. Challenges were specifically a lack of knowledge and training in managing comorbidities and subsequently the concern regarding the consequences for the patients. Further investigations confirming the findings and including healthcare professions other than physiotherapists and occupational therapists are warranted. Furthermore, additional literature related to physiotherapists and occupational therapists will also be necessary to substantiate the findings of this review.

## Author Contributions

L.C.R., J.S.G., T.S.P., S.T.S. and J.A. conceived the conceptualisation and design of the study. L.C.R. and J.S.G. were responsible for performing the search. L.C.R. and J.S.G. were responsible for the selection of studies and L.C.R. for the critical appraisal. L.C.R. and T.S.P. drafted the manuscript. J.S.G., J.A. and S.T.S. revised the article critically for important intellectual content. All authors participated in the revision and final approval of the manuscript.

## Ethics Statement

This study is a scoping review, which involves the analysis of existing literature and does not involve the direct participation of human subjects. Consequently, it does not require approval from the Research Ethics Committee.

## Conflicts of Interest

The authors declare no conflicts of interest.

## Supporting information


**Data S1:** scs70130‐sup‐0001‐Supinfo01.docx.

**Data S2:** scs70130‐sup‐0002‐Supinfo02.docx.

**Data S3:** scs70130‐sup‐0003‐Supinfo03.docx.

**Data S4:** scs70130‐sup‐0004‐Supinfo04.docx.

**Data S5:** scs70130‐sup‐0005‐Supinfo05.docx.

**Data S6:** scs70130‐sup‐0006‐Supinfo06.docx.

## Data Availability

Data sharing is not applicable to this article as no new data were created or analysed in this study.

## References

[1] S. T. Skou , F. S. Mair , M. Fortin , et al., “Multimorbidity,” Nature Reviews. Disease Primers 8, no. 1 (2022): 48. 10.1038/s41572-022-00376-4 PMC7613517 35835758

[2] D. B. Lowe , M. J. Taylor , and S. J. Hill , “Cross‐Sectional Examination of Musculoskeletal Conditions and Multimorbidity: Influence of Different Thresholds and Definitions on Prevalence and Association Estimates,” BMC Research Notes 10, no. 1 (2017): 1–13. 28100264 10.1186/s13104-017-2376-4 PMC5242059

[3] A. Van Der Zee‐Neuen , P. Putrik , S. Ramiro , et al., “Work Outcome in Persons With Musculoskeletal Diseases: Comparison With Other Chronic Diseases & the Role of Musculoskeletal Diseases in Multimorbidity,” BMC Musculoskeletal Disorders 18, no. 1 (2017): 1–8. 28069020 10.1186/s12891-016-1365-4 PMC5223391

[4] S. J. Duffield , B. M. Ellis , N. Goodson , et al., “The Contribution of Musculoskeletal Disorders in Multimorbidity: Implications for Practice and Policy,” Best Practice & Research. Clinical Rheumatology 31, no. 2 (2017): 129–144. 29224692 10.1016/j.berh.2017.09.004

[5] P. E. Muckelt , E. Roos , M. Stokes , et al., “Comorbidities and Their Link With Individual Health Status: A Cross‐Sectional Analysis of 23,892 People With Knee and Hip Osteoarthritis From Primary Care,” Journal of Comorbidity 10 (2020): 2235042X2092045. 10.1177/2235042X20920456 PMC7238776 32489945

[6] B. Guthrie , K. Payne , P. Alderson , M. E. T. McMurdo , and S. W. Mercer , “Adapting Clinical Guidelines to Take Account of Multimorbidity,” BMJ (Clinical Research Ed.) 345, no. 1 (2012): e6341. 10.1136/bmj.e6341 23036829

[7] M. Kastner , L. Hayden , G. Wong , et al., “Underlying Mechanisms of Complex Interventions Addressing the Care of Older Adults With Multimorbidity: A Realist Review,” BMJ Open 9, no. 4 (2019): 1–11. 10.1136/bmjopen-2018-025009 PMC6500199 30948577

[8] J. He , D. R. Morales , and B. Guthrie , “Exclusion Rates in Randomized Controlled Trials of Treatments for Physical Conditions: A Systematic Review,” Trials 21, no. 1 (2020): 228. 32102686 10.1186/s13063-020-4139-0 PMC7045589

[9] L. D. Hughes , M. E. T. McMurdo , and B. Guthrie , “Guidelines for People Not for Diseases: The Challenges of Applying UK Clinical Guidelines to People With Multimorbidity,” Age and Ageing 42, no. 1 (2013): 62–69. 22910303 10.1093/ageing/afs100

[10] A. F. Pedersen , P. Vedsted , and K. B. Nørøxe , “Influence of Patient Multimorbidity on GP Burnout: A Survey and Register‐Based Study in Danish General Practice,” British Journal of General Practice 70, no. 691 (2020): E95–E101. 10.3399/bjgp20X707837 PMC6960003 31932298

[11] E. Sondergaard , T. G. Willadsen , A. D. Guassora , et al., “Problems and Challenges in Relation to the Treatment of Patients With Multimorbidity: General Practitioners' Views and Attitudes,” Scandinavian Journal of Primary Health Care 33, no. 2 (2015): 121–126. 26158584 10.3109/02813432.2015.1041828 PMC4834499

[12] Y. Fu , A. Chiarotto , W. Enthoven , S. T. Skou , and B. Koes , “The Influence of Comorbidities on Outcomes for Older People With Back Pain: BACE‐D Cohort Study,” Annals of Physical and Rehabilitation Medicine 66, no. 7 (2023): 101754. 37276834 10.1016/j.rehab.2023.101754

[13] R. A. Damarell , D. D. Morgan , and J. J. Tieman , “General Practitioner Strategies for Managing Patients With Multimorbidity: A Systematic Review and Thematic Synthesis of Qualitative Research,” BMC Family Practice 21, no. 1 (2020): 131. 32611391 10.1186/s12875-020-01197-8 PMC7331183

[14] J. Thomas and A. Harden , “Methods for the Thematic Synthesis of Qualitative Research in Systematic Reviews,” BMC Medical Research Methodology 8, no. 1 (2008): 45. 18616818 10.1186/1471-2288-8-45 PMC2478656

[15] Critical Appraisal Skills Programme , CASP Qualitative Studies Checklist 2018.

[16] H. A. Long , D. P. French , and J. M. Brooks , “Optimising the Value of the Critical Appraisal Skills Programme (CASP) Tool for Quality Appraisal in Qualitative Evidence Synthesis,” Research Methods in Medicine & Health Sciences 1, no. 1 (2020): 31–42.

[17] A. J. Gibbs , J. A. Wallis , N. F. Taylor , J. L. Kemp , and C. J. Barton , “Osteoarthritis Management Care Pathways Are Complex and Inefficient: A Qualitative Study of Physiotherapist Perspectives From Specialised Osteoarthritis Services,” Musculoskeletal Care 20, no. 4 (2022): 860–872. 35403316 10.1002/msc.1638 PMC10084427

[18] L. Hemmings and A. Soundy , “Experiences of Physiotherapy in Mental Health: An Interpretative Phenomenological Analysis of Barriers and Facilitators to Care,” Physiotherapy 109 (2020): 94–101. 32522361 10.1016/j.physio.2020.01.001

[19] L. K. King , E. J. Waugh , C. MacKay , et al., “Formulating Knee Osteoarthritis Management Plans Taking Type 2 Diabetes Into Account: Qualitative Study of Arthritis Therapists Using Theoretical Domains Framework,” Journal of Rheumatology 49, no. 12 (2022): 1365–1371. 36109079 10.3899/jrheum.220535

[20] B. J. Lawford , K. L. Bennell , K. Allison , S. Schwartz , and R. S. Hinman , “Challenges With Strengthening Exercises for Individuals With Knee Osteoarthritis and Comorbid Obesity: A Qualitative Study With Patients and Physical Therapists,” Arthritis Care and Research 74, no. 1 (2022): 113–125. 32886868 10.1002/acr.24439

[21] P. L. Teo , K. L. Bennell , B. J. Lawford , T. Egerton , K. S. Dziedzic , and R. S. Hinman , “Physiotherapists May Improve Management of Knee Osteoarthritis Through Greater Psychosocial Focus, Being Proactive With Advice, and Offering Longer‐Term Reviews: A Qualitative Study,” Journal of Physiotherapy 66, no. 4 (2020): 256–265. 33036932 10.1016/j.jphys.2020.09.005

[22] M. Rosbach and J. S. Andersen , “Patient‐Experienced Burden of Treatment in Patients With Multimorbidity – A Systematic Review of Qualitative Data,” PLoS One 12, no. 6 (2017): e0179916–e0179916. 28644877 10.1371/journal.pone.0179916 PMC5482482

[23] M. Jäger , M. C. Lindhardt , J. R. Pedersen , et al., “Putting the Pieces Together: A Qualitative Study Exploring Perspectives on Self‐Management and Exercise Behavior Among People Living With Multimorbidity, Healthcare Professionals, Relatives, and Patient Advocates,” Journal of Multimorbidity and Comorbidity 12 (2022): 263355652211001. 10.1177/26335565221100172 PMC9125109 35615752

[24] A. Synnott , M. O'Keeffe , S. Bunzli , W. Dankaerts , P. O'Sullivan , and K. O'Sullivan , “Physiotherapists May Stigmatise or Feel Unprepared to Treat People With Low Back Pain and Psychosocial Factors That Influence Recovery: A Systematic Review,” Journal of Physiotherapy 61, no. 2 (2015): 68–76. 25812929 10.1016/j.jphys.2015.02.016

[25] C. Driver , B. Kean , F. Oprescu , and G. P. Lovell , “Knowledge, Behaviors, Attitudes and Beliefs of Physiotherapists Towards the Use of Psychological Interventions in Physiotherapy Practice: A Systematic Review,” Disability and Rehabilitation 39, no. 22 (2017): 2237–2249. 27635464 10.1080/09638288.2016.1223176

[26] J. S. Gandløse , T. F. Tróndarson , J. Vela , et al., “Does Spinal Pain Management Account for the Presence of Other Health Conditions? – A Scoping Review,” European Journal of Physiotherapy 27 (2024): 248–258.

[27] T. McIntosh , D. J. Hunter , and S. Royce , “Barriers to Physical Activity in Obese Adults: A Rapid Evidence Assessment,” Journal of Research in Nursing 21, no. 4 (2016): 271–287.

[28] J. Sallinen , R. Leinonen , M. Hirvensalo , T. M. Lyyra , E. Heikkinen , and T. Rantanen , “Perceived Constraints on Physical Exercise Among Obese and Non‐Obese Older People,” Preventive Medicine 49, no. 6 (2009): 506–510. 19833148 10.1016/j.ypmed.2009.10.001

[29] J. Setchell , B. Watson , L. Jones , and M. Gard , “Weight Stigma in Physiotherapy Practice: Patient Perceptions of Interactions With Physiotherapists,” Manual Therapy 20, no. 6 (2015): 835–841. 25920342 10.1016/j.math.2015.04.001

[30] F. G. Schellevis , J. van der Velden , E. van de Lisdonk , J. T. M. van Eijk , and C. van Weel , “Comorbidity of Chronic Diseases in General Practice,” Journal of Clinical Epidemiology 46, no. 5 (1993): 469–473. 8501473 10.1016/0895-4356(93)90024-u

[31] M. Blagojevic , C. Jinks , A. Jeffery , and K. P. Jordan , “Risk Factors for Onset of Osteoarthritis of the Knee in Older Adults: A Systematic Review and Meta‐Analysis,” Osteoarthritis and Cartilage 18, no. 1 (2010): 24–33. 19751691 10.1016/j.joca.2009.08.010

[32] NICE , “Chronic Pain (Primary and Secondary) in Over 16s: Assessment of All Chronic Pain and Management of Chronic Primary Pain NICE Guideline [Internet],” (2021), www.nice.org.uk/guidance/ng193. 33939353

[33] A. Bricca , L. K. Harris , M. Jäger , S. M. Smith , C. B. Juhl , and S. T. Skou , “Benefits and Harms of Exercise Therapy in People With Multimorbidity: A Systematic Review and Meta‐Analysis of Randomised Controlled Trials,” Ageing Research Reviews 63 (2020): 101166. 32896665 10.1016/j.arr.2020.101166 PMC7116122

[34] B. K. Pedersen and B. Saltin , “Exercise as Medicine ‐ Evidence for Prescribing Exercise as Therapy in 26 Different Chronic Diseases,” Scandinavian Journal of Medicine & Science in Sports 25 (2015): 1–72. 10.1111/sms.12581 26606383

[35] S. T. Skou , M. Nyberg , M. Dideriksen , et al., “Study Protocol for a Multicenter Randomized Controlled Trial of Personalized Exercise Therapy and Self‐Management Support for People With Multimorbidity: The Mobilize Study,” Journal of Multimorbidity and Comorbidity 13 (2023): 263355652311544. 10.1177/26335565231154447 PMC9903016 36762033

