# Supplementary material for: Challenges and Perspectives in Treating Individuals With Musculoskeletal Disorders and Comorbidity: A Systematic Literature Review With a Descriptive Thematic Synthesis
Source: Scand J Caring Sci. 2025 Oct 3;39(4):e70130. doi: 10.1111/scs.70130 (PMC12495375; doi:10.1111/scs.70130)
Supplement: Supplementary file 2 — Data S2: scs70130‐sup‐0002‐Supinfo02.docx. [file SCS-39-0-s003.docx]

## Appendix 2: Search protocol

|  | AND | | | |
| --- | --- | --- | --- | --- |
|  | **Musculoskeletal disorders** | **Multimorbidity/Comorbidity** | **Healthcare professionals** | **Qualitative** |
| OR | Musculoskeletal pain  Musculoskeletal disorders  Musculoskeletal conditions  Musculoskeletal diseases  Rheumatic condition  Rheumatic diseases  Skeletal disorders  Skeletal conditions  Orthopedic disorders  Orthopedic conditions  Orthopedic diseases  Diseases of the musculoskeletal system  Spinal pain  Back pain  Knee pain  Shoulder pain  Arthritis  Osteoarthritis  Osteoporosis  Gout | Comorbidity  Co-morbidity  Multimorbidity  Multi-morbidity  Comorbidities  Multiple health problems  Dual diagnosis  Coexisting conditions  Multiple health conditions  Multiple morbidity  Multiple morbidities  Multiple conditions  Multi disease  Multiple abnormalities  Diagnoses AND multiple:  (Diabetes mellitus, Hypertension, Heart Diseases, Cerebrovascular disorders, Asthma, Pulmonary disease chronic obstructive, Hyperlipidemia, Thyroid diseases, Arthritis rheumatoid, Mental disorders, Epilepsy, Hiv infections, Neoplasms, Kidney diseases, Liver diseases, Osteoporosis AND coocur or co-occur or coexist or multiple) | General practitioner  Physiotherapist  Nurse  Doctor  Orthopedic surgeon  Healthcare professionals  Medical professionals  Health professionals  Healthcare providers  Healthcare workers  Health practitioners  Health providers  Health workers  Healthcare practitioners  Clinical personnel  Osteopath  Providers  Physician  Rheumatologist | Qualitative research  Qualitative evaluation  Qualitative study  Qualitative design  Qualitative  Narrative  Qualitative method  Interview  Qualitative data  Grounded Theory  Phenomenological study  Narrative analysis  Narratives  Experience  Perspective  View  Perception  Attitude |
