# Supplementary material for: Challenges and Perspectives in Treating Individuals With Musculoskeletal Disorders and Comorbidity: A Systematic Literature Review With a Descriptive Thematic Synthesis
Source: Scand J Caring Sci. 2025 Oct 3;39(4):e70130. doi: 10.1111/scs.70130 (PMC12495375; doi:10.1111/scs.70130)
Supplement: Supplementary file 3 — Data S3: scs70130‐sup‐0003‐Supinfo03.docx. [file SCS-39-0-s006.docx]

## Appendix 3: Study design, aim, population, methods, results and conclusions of the included studies

| **Study 1: Gibbs et al., [17]** | |
| --- | --- |
| Study design | Qualitative study |
| Study aim | To investigate physiotherapist perspectives of osteoarthritis care. |
| Population | Physiotherapists from specialized osteoarthritis services |
| Methods | Nineteen semi-structured interviews were conducted with physiotherapists working in specialist osteoarthritis services across three different Australian models of care. |
| Results | The overarching theme to emerge was that osteoarthritis care is complex and difficult regardless of model of care. Subthemes indicated that: services are either unavailable or inadequately funded; referral pathways are labyrinthine and lengthy; patients and other health professionals often believe that surgery is the only /best option and; managing patient comorbidities is challenging.  Managing patient comorbidities: The findings highlight additional challenges in managing osteoarthritis in people with comorbidities such as obesity and depression. Frustration with an apparent lack of effective strategies for weight management was expressed, while acknowledging it is complex and often difficult for patients to lose weight. |
| Conclusion | Physiotherapists working in specified osteoarthritis services perceive multiple barriers influencing adherence to care. Barriers occur at various levels in all models of care, and improving healthcare for people with osteoarthritis requires urgent system reform. |
| **Study 2: Hemmings & Soundy, [18]** | |
| Study design | Qualitative study. |
| Study aim | To explore experiences of Physiotherapeutic care for those with comorbid physical and mental health complaints to identify barriers and facilitators to care. |
| Population | Mental health physiotherapists and individuals diagnosed with severe mental illness (SMI) alongside a comorbid physical health complaint requiring physiotherapeutic input. |
| Methods | Semi-structured interviews were completed with service users with longstanding physiotherapeutic and psychiatric complaints. Focus groups were completed with physiotherapists working in mental health. The transcripts were analyzed using Interpretive Phenomenological analysis. |
| Results | The most common mental health diagnosis was psychosis (n=3/8) and the most common physiotherapy complaint was chronic back pain (n=3/8). Certain aspects within physiotherapy sessions were of great importance to achieving this positive experience for the service user. These factors included: (1) patient-therapist interaction, (2) holistic approach to care, (3) therapist awareness and experience of mental health. Physiotherapists partaking in focus groups suggested that a lack of education and experience within mental health specialties was partly responsible for minimal consideration of the psychosocial aspects of care |
| Conclusion | This study confirms a lack of mental health awareness among physiotherapists outside their specialty. All physiotherapists should be confident in discussing mental health with patients, especially due to the close relationships between pain, disability, and mental well-being. Enhanced education is needed at both undergraduate and postgraduate levels to ensure physiotherapists feel self-assured when treating patients with mental disorders. |
| **Study 3: King et al., [19]** | |
| Study design | Qualitative study |
| Study aim | To understand the extent to which arthritis therapists (Physical therapists and Occupational therapists) consider type 2 diabetes mellitus (T2DM) when treating persons with knee OA and comorbid T2DM, and barriers to doing so. |
| Population | Arthritis therapists working within a provincially funded arthritis care program (Arthritis Society Canada) in Ontario, Canada. |
| Methods | 18 semi structured telephone interviews with arthritis therapists were interviewed. The interviews were analyzed using the Theoretical Domains Framework (TDF) to identity barriers and enablers. Within TDF domains themes were inductively devolved. |
| Results | Five TDF domains significantly influencing the behavior of arthritis therapists when developing knee osteoarthritis management plans in consideration of concurrent type 2 diabetes were identified. These domains include therapists' perceived lack of specific knowledge regarding comorbidities, particularly diabetes; limited skills in behavioral change techniques for assisting patients with goal-setting, especially related to physical activity; inconsistent intention to incorporate a patient's comorbidity profile into treatment recommendations; a perception of their professional identity as joint-focused; and the environmental context. |
| Conclusion | Within the context of a Canadian arthritis program several barriers to arthritis therapists considering T2DM in their management plan for persons with knee OA and T2DM were identified. |
| **Study 4: Lawford et al., [20]** | |
| Study design | Qualitative study. |
| Study aim | To explore challenges associated with implementing a home-based strengthening exercise program for individuals with knee osteoarthritis and comorbid obesity. |
| Population | Patients with knee osteoarthritis and comorbid obesity and physiotherapists working with these patients. |
| Methods | This qualitative research is part of a larger randomized controlled trial that compares two home-based strengthening programs: one involving weight-bearing functional exercises and the other focusing on non-weight-bearing quadriceps strengthening exercises, intended for individuals dealing with knee osteoarthritis and coexisting obesity. Participants in both exercise programs attended five consultations with a physical therapist and followed a home-based exercise routine for a duration of 12 weeks. Following the completion of the trial, semi structured individual telephone interviews were conducted with 22 patients and all seven physical therapists who administered the trial interventions. These interviews were recorded, transcribed verbatim, and subsequently analyzed thematically using an inductive approach. |
| Results | Three overarching themes emerged from the study's findings: Psychological challenges; physical challenge; and overcoming challenges. Psychological challenges encompassed misconceptions about exercise, a fear of experiencing pain, a general lack of enthusiasm for engaging in physical activity, the mental effort required for the weight-bearing functional program, and the tendency to underestimate one's own capabilities. Physical challenges included the perceived complexity of the weight-bearing functional program, difficulties encountered with cuff weights and straight leg raises in the non-weight-bearing quadriceps program, as well as considerations related to other pre-existing health conditions. Finally, the study revealed strategies for overcoming these challenges, where participants highlighted the importance of incentives to motivate exercise, the need for accountability to maintain a consistent exercise routine, the value of education and reassurance regarding the exercise regimen, and the customization of the exercise program to better suit individual needs. |
| Conclusion | We found that patients and physical therapists experienced numerous psychological and physical challenges to exercise. |
| **Study 5: Teo et al., [21]** | |
| Study design | Qualitative study. |
| Study aim | To investigate physiotherapist perspectives of delivering care for people with knee osteoarthritis. |
| Population | Twenty-two physiotherapists with experience in providing care for people with knee osteoarthritis. |
| Methods | Semi-structured individual telephone interviews were conducted. Questions were informed by seven quality statements from the national Knee Osteoarthritis Clinical Care Standard. Thematic analysis was undertaken, with themes/subthemes inductively derived. Interview data were also deductively analyzed according to the Clinical Care Standard. |
| Results | Five themes emerged: Biomedical management with an aim to improve function; Personalized program with exercise at the heart of physiotherapists’ care; Clinical challenges; Knowing versus doing; It’s not my job.  Clinical challenges included patient comorbidity. Comorbidities (e.g., overweight and obesity, cardiovascular disease, fibromyalgia, rheumatoid arthritis, complex regional pain syndrome) were described as a barrier to evidence-based management. Physio-therapists felt that patients with comorbidities often experienced more severe pain, hampering their ability to exercise or be physically active. Some physiotherapists found it difficult to manage patients with comorbid obesity.  The theme “It’s not my job” stems from the fact that physiotherapists considered some elements of OA care to be outside their scope of practice, particularly weight loss, medication and surgical advice. Although most tended to mention weight loss in management discussions with patients who were overweight or obese, they felt that this aspect of the care was the role of a dietician. They favored referring patients who needed to lose weight to other healthcare providers. |
| Conclusion | Physiotherapists reported care for people with knee osteoarthritis that was mostly consistent with the quality care standard. Care may be improved by increasing the psychosocial focus of care, offering longer-term reviews, and being more proactive with advice and/or referral regarding weight loss, pain medications and knee surgery. |
