# Supplementary material for: Challenges and Perspectives in Treating Individuals With Musculoskeletal Disorders and Comorbidity: A Systematic Literature Review With a Descriptive Thematic Synthesis
Source: Scand J Caring Sci. 2025 Oct 3;39(4):e70130. doi: 10.1111/scs.70130 (PMC12495375; doi:10.1111/scs.70130)
Supplement: Supplementary file 4 — Data S4: scs70130‐sup‐0004‐Supinfo04.docx. [file SCS-39-0-s001.docx]

## Appendix 4: Search strategy for all the included databases

| Search strategy in Pubmed | | | | | | |
| --- | --- | --- | --- | --- | --- | --- |
| **Musculoskeletal disorders** | **Multimorbidity/Comorbidity** | | **Healthcare professionals** | | | **Qualitative** |
| Thesaurus Terms (Mesh) | | | | | | |
| Musculoskeletal pain  Musculoskeletal abnormalities  Musculoskeletal disease | Comorbidities  Multiple abnormalities | | Health Personnel  Physiotherapist  Nurse clinician  Doctor of osteopathy  Health care provider | | | Qualitative research  Qualitative evaluation  Interview  Narration  Personal Narratives |
| Free text (Title/Abstract) | | | | | | |
| Musculoskeletal*  Rheumatic*  Skeletal*  Orthopedic*  “Diseases of the musculoskeletal system”  “Spinal pain”  “Back pain”  “Knee pain”  “Shoulder pain”  Arthritis  Osteoarthritis  Osteoporosis  Gout | Comorbidit*  Co-morbidit*  Multimorbidit*  Multi-morbidit*  “Multiple health condition*”  “Multiple morbidit*”  “Multiple health problem*”  “Dual diagnoses”  “Coexisting condition*”  “Multiple health condition*”  “Multiple condition*”  “Multiple abnormalities”  Diagnoses AND multiple:  (“Diabetes mellitus” or Hypertension or “Heart Disease*” or “Cerebrovascular disorder*” or Asthma or “Pulmonary disease chronic obstructive” or, Hyperlipidemia or “Thyroid diseases” or “ Arthritis rheumatoid” or “Mental disorders” or Epilepsy or “Hiv infections” or Neoplasms or “Kidney diseases” or “ Liver diseases” or Osteoporosis) AND (co-oocur or coexist or multiple) | | “General practitioner*”  Physiotherapist*  Nurse*  Doctor*  “Orthopedic surgeon*”  “Healthcare professional*”  “Medical professional*”  “Health professional”  “Healthcare provider*”  “Healthcare worker*”  “Health provider*”  “Health worker*”  “Health practitioner*”  “Healthcare practitioner*”  “Clinical personnel*”  Osteopath*  Provider*  Physician*  Rheumatologist*  * | | | “Interview*”  “Qualitativ*”  narrative*  view*  perspective*  experience* |
| Results: 1.164.930 | | Results: 468.943 | | Results: 1.573.314 | Results: 3.879.733 | |
| Filters: Language (English, German, Danish, Norwegian, Swedish) + full text + NOT (Review or Systematic review)  Results in total: 813  Date of search: 04-10-23 | | | | | | |
| Search strategy in Cinahl | | | | | | |
| **Musculoskeletal disorders** | **Multimorbidity/Comorbidity** | | **Healthcare professionals** | | | **Qualitative** |
| Thesaurus Terms (Subheadings) | | | | | | |
| Musculoskeletal pain  Musculoskeletal abnormalities  Musculoskeletal disease  Rheumatology | Comorbidity | | Health Personnel  Physical Therapists  Nurses | | | Qualitative studies  Interviews  Narratives |
| Free Text (Title/Abstract) | | | | | | |
| Musculoskeletal*  Rheumatic*  Skeletal*  Orthopedic*  “Diseases of the musculoskeletal system”  “Spinal pain”  “Back pain”  “Knee pain”  “Shoulder pain”  Arthritis  Osteoarthritis  Osteoporosis  Gout | Comorbidit*  Co-morbidit*  Multimorbidit*  Multi-morbidit*  “Multiple health condition*”  “Multiple morbidit*”  “Multiple health problem*”  “Dual diagnoses”  “Coexisting condition*”  “Multiple health condition*”  “Multiple condition*”  “Multiple abnormalities”  Diagnoses AND multiple:  (“Diabetes mellitus” or Hypertension or “Heart Diseases” or “Cerebrovascular disorders” or Asthma or “Pulmonary disease chronic obstructive” or, Hyperlipidemia or “Thyroid diseases” or “ Arthritis rheumatoid” or “Mental disorders” or Epilepsy or “Hiv infections” or Neoplasms or “Kidney diseases” or “ Liver diseases” or Osteoporosis) AND (coocur or co-occur or coexist or multiple) | | “General practitioner*”  Physiotherapist*  Nurse*  Doctor*  “Orthopedic surgeon*”  “Healthcare professional*”  “Medical professional*”  “Health professional”  “Healthcare provider*”  “Healthcare worker*”  “Health provider*”  “Health worker*”  “Health practitioner*”  “Healthcare practitioner*”  “Clinical personnel*”  Osteopath  Provider*  Physician*  Rheumatologist*  * | | | “Interview*”  “Qualitativ*”  narrative*  view*  perspective*  experience* |
| Results: 485.822 | Results: 113.345 | | Results: 1.014.225 | | | Results: 1.009.558 |
| Filter: NOT (Review or Systematic review) + language (English, German, Danish, Norwegian, Swedish) + full text  Results in total: 22  Date of search: 04-10-23 | | | | | | |
| Search strategy in Embase | | | | | | |
| **Musculoskeletal disorders** | **Multimorbidity/Comorbidity** | | **Healthcare professionals** | | | **Qualitative** |
| Thesaurus (Mesh) | | | | | | |
| Musculoskeletal system  Musculoskeletal disease  Rheumatology | Comorbidity  Multiple chronic conditions | | Health care personnel  Physiotherapist  Nurse | | | Qualitative research  Interviews  Narrative |
| Free Text (Title/Abstract) | | | | | | |
| Musculoskeletal*  Rheumatic*  Skeletal*  Orthopedic*  “Diseases of the musculoskeletal system”  “Spinal pain”  “Back pain”  “Knee pain”  “Shoulder pain”  Arthritis  Osteoarthritis  Osteoporosis  Gout | Comorbidit*  Co-morbidit*  Multimorbidit*  Multi-morbidit*  “Multiple health condition*”  “Multiple morbidit*”  “Multiple health problem*”  “Dual diagnoses”  “Coexisting condition*”  “Multiple health condition*”  “Multiple condition*”  “Multiple abnormalities”  Diagnoses AND multiple:  (“Diabetes mellitus” or Hypertension or “Heart Diseases” or “Cerebrovascular disorders” or Asthma or “Pulmonary disease chronic obstructive” or, Hyperlipidemia or “Thyroid diseases” or “ Arthritis rheumatoid” or “Mental disorders” or Epilepsy or “Hiv infections” or Neoplasms or “Kidney diseases” or “ Liver diseases” or Osteoporosis) AND (coocur or co-occur or coexist or multiple) | | “General practitioner*”  Physiotherapist*  Nurse*  Doctor*  “Orthopedic surgeon*”  “Healthcare professional*”  “Medical professional*”  “Health professional”  “Healthcare provider*”  “Healthcare worker*”  “Health provider*”  “Health worker*”  “Health practitioner*”  “Healthcare practitioner*”  “Clinical personnel*”  Osteopath  Provider*  Physician*  Rheumatologist*  * | | | “Interview*”  “Qualitativ*”  narrative*  view*  perspective*  experience* |
| Results: 4.696.184 | Results: 756.465 | | Results: 2.838.656 | | | Results: 3.758.135 |
| Filter: NOT (Review or Systematic review)) + language (English, German, Danish, Norwegian, Swedish) + full text + NOT conference abstracts  Results in total: 1.876  Date of search: 04-10-23 | | | | | | |
| Search strategy in Scopus | | | | | | |
| **Musculoskeletal disorders** | **Multimorbidity/Comorbidity** | | **Healthcare professionals** | | | **Qualitative** |
| Free Text (Title/Abstract/Keywords) | | | | | | |
| Musculoskeletal*Rheumatic*Skeletal*Orthopedic*“Diseases of the musculoskeletal system”“Spinal pain”“Back pain”“Knee pain”“Shoulder pain”ArthritisOsteoarthritisOsteoporosisGout | Comorbidit*  Co-morbidit*  Multimorbidit*  Multi-morbidit*  “Multiple health condition*”  “Multiple morbidit*”  “Multiple health problem*”  “Dual diagnoses”  “Coexisting condition*”  “Multiple health condition*”  “Multiple condition*”  “Multiple abnormalities”  Diagnoses AND multiple:  (“Diabetes mellitus” or Hypertension or “Heart Diseases” or “Cerebrovascular disorders” or Asthma or “Pulmonary disease chronic obstructive” or, Hyperlipidemia or “Thyroid diseases” or “ Arthritis rheumatoid” or “Mental disorders” or Epilepsy or “Hiv infections” or Neoplasms or “Kidney diseases” or “ Liver diseases” or Osteoporosis) AND (coocur or co-occur or coexist or multiple) | | “General practitioner*”  Physiotherapist*  Nurse*  Doctor*  “Orthopedic surgeon*”  “Healthcare professional*”  “Medical professional*”  “Health professional”  “Healthcare provider*”  “Healthcare worker*”  “Health provider*”  “Health worker*”  “Health practitioner*”  “Healthcare practitioner*”  “Clinical personnel*”  Osteopath  Provider*  Physician*  Rheumatologist*  * | | | “Interview*”  “Qualitativ*”  narrative*  view*  perspective*  experience* |
| Results: 1.576.202 | Results: 946.013 | | Results: 2.162.074 | | | Results: 7.999.162 |
| Filter: NOT (Review or Systematic review) + language (English, German, Danish, Norwegian, Swedish) + full text  Results in total: 1.188  Date of search: 04-10-23 | | | | | | |
