# Supplementary material for: Challenges and Perspectives in Treating Individuals With Musculoskeletal Disorders and Comorbidity: A Systematic Literature Review With a Descriptive Thematic Synthesis
Source: Scand J Caring Sci. 2025 Oct 3;39(4):e70130. doi: 10.1111/scs.70130 (PMC12495375; doi:10.1111/scs.70130)
Supplement: Supplementary file 5 — Data S5: scs70130‐sup‐0005‐Supinfo05.docx. [file SCS-39-0-s005.docx]

## Appendix 5: Excluded studies after full text screening

| **Reason for exclusion** | **Title** |
| --- | --- |
| Wrong scope | ‘Local adaptation of recommendation-based materials for shared decision-making and management of comorbidity in rheumatoid arthritis.’ [1] |
|  | ‘Impact of comorbidity’ [2] |
|  | ‘Priorities for high-quality care in rheumatoid arthritis: results of patient, health professional, and policy maker perspectives’ [3] |
|  | ‘Redesigning care for chronic conditions: improving hospital-based ambulatory care for people with osteoarthritis of the hip and knee’ [4] |
|  | ‘Capacity, responsibility, and motivation: A critical qualitative evaluation of patient and practitioner views about barriers to self-management in people with multimorbidity’ [5] |
|  | ‘Primary care practitioner and patient understanding of the concepts of multimorbidity and self-management: A qualitative study’ [6] |
|  | ‘Barriers and enablers to health care providers’ assessment and treatment of knee osteoarthritis in persons with type 2 diabetes mellitus: A qualitative study using the Theoretical Domains Framework’ [7] |
|  | ‘Multimorbidity from the general practitioner's perspective’ [8] |
|  | ‘Managing complex medication regimens: perspectives of consumers with osteoarthritis and healthcare professionals’ [9] |
|  | ‘Treatment burden among people with chronic illness: what are consumer health organizations saying?’ [10] |
|  | ‘'Trying to put a square peg into a round hole': a qualitative study of healthcare professionals' views of integrating complementary medicine into primary care for musculoskeletal and mental health comorbidity’ [11] |
|  | ‘Clinicians' perspectives of shared care of psoriatic arthritis and psoriasis between rheumatology and dermatology: an interview study’ [12] |
|  | ‘Migraine and Central Sensitization: Clinical Features, Main Comorbidities and Therapeutic Perspectives’ [13] |
|  | ‘Evaluation of an educational course for primary care physiotherapists on comorbidity-adapted exercise therapy in knee osteoarthritis: an observational study’ [14] |
|  | ‘Rheumatologists' Views and Experiences in Managing Rheumatoid Arthritis in Elderly Patients: A Qualitative Study’ [15] |
| Wrong study design | ‘Integrated headache care: Experiences from Germany, Denmark, and USA’ [16] |
|  | ‘Opening the black box of psychological treatments for chronic pain: A clinical perspective for medical providers’ [17] |
|  | ‘Comorbidity in rheumatic diseases’ [18] |
|  | ‘Established rheumatoid arthritis: Rationale for best practice - Physicians' perspective of how to realise tight control in clinical practice’ [19] |
|  | ‘Considerations for improving quality of care of patients with rheumatoid arthritis and associated comorbidities’ [20] |
|  | ‘Physiotherapist beliefs and perspectives on virtual reality-supported rehabilitation for the assessment and management of musculoskeletal shoulder pain: a focus group study protocol’ [21] |
|  | ‘Consensus statement on a framework for the management of comorbidity and extra-articular manifestations in rheumatoid arthritis’ [22] |
|  | ‘Chronic pain and multimorbidity: Comment on "Chronic pain education in Portugal: Perspectives from medical students and interns”’ [23] |
|  | ‘Managing multimorbidity in primary care in patients with chronic respiratory conditions’ [24] |
|  | ‘How to improve care for patients with RA and comorbidities’ [25] |
|  | ‘Improving the experiences and health of people with multimorbidity: exploratory research with policymakers and information providers on comorbid arthritis’ [26] |
|  | ‘Clinical experience of rehabilitation therapists with chronic diseases: a quantitative approach’ [27] |
| Wrong population | ‘A qualitative study of perceived needs and factors associated with the quality of care for common mental disorders in patients with chronic diseases: the perspective of primary care clinicians and patients’ [28] |
|  | ‘How do middle-aged patients and their healthcare providers manage multimorbidity? Results of a qualitative study’ [29] |
|  | ‘Barriers to accessing health care for people with chronic conditions: a qualitative interview study’ [30] |
|  | ‘Perceptions, attitudes and training needs of primary healthcare professionals in identifying and managing frailty: a qualitative study’ [31] |
|  | ‘Exploring the facilitators, barriers, and strategies for self-management in adults living with severe mental illness, with and without long-term conditions: A qualitative evidence synthesis’ [32] |
|  | ‘Barriers and facilitators to chronic pain self-management: a qualitative study of primary care patients with comorbid musculoskeletal pain and depression’ [33] |

**References**

1. Álvaro Gracia JM, Barbazán C, García Llorente JF, Muñóz-Fernández S, Gómez Centeno A, Urruticoechea-Arana A, et al. Local adaptation of recommendation-based materials for shared decision-making and management of comorbidity in rheumatoid arthritis. Clin Exp Rheumatol. 2021/07/13 ed. 2022 May;40(5):975–9.

2. Azeez M, Taylor PC. Impact of comorbidity. In: Comorbidity in Rheumatic Diseases [Internet]. 2017. p. 33–52. Available from: https://www.scopus.com/inward/record.uri?eid=2-s2.0-85055365950&doi=10.1007%2f978-3-319-59963-2_2&partnerID=40&md5=2e5bf8396d3ca604487eba5173623a63

3. Barber CEH, Lacaille D, Hall M, Bohm V, Li LC, Barnabe C, et al. Priorities for high-quality care in rheumatoid arthritis: results of patient, health professional, and policy maker perspectives. Journal of Rheumatology. 2021;48(4):486–94.

4. Brand CA, Amatya B, Gordon B, Tosti T, Gorelik A. Redesigning care for chronic conditions: improving hospital-based ambulatory care for people with osteoarthritis of the hip and knee. Intern Med J. 2009/03/28 ed. 2010 Jun;40(6):427–36.

5. Coventry PA, Fisher L, Kenning C, Bee P, Bower P. Capacity, responsibility, and motivation: A critical qualitative evaluation of patient and practitioner views about barriers to self-management in people with multimorbidity. BMC Health Services Research [Internet]. 2014;14(1). Available from: https://www.scopus.com/inward/record.uri?eid=2-s2.0-84920837339&doi=10.1186%2fs12913-014-0536-y&partnerID=40&md5=34bd3c862c0bc9ca1cf6a283d758a2cf

6. Kenning C, Fisher L, Bee P, Bower P, Coventry P. Primary care practitioner and patient understanding of the concepts of multimorbidity and self-management: A qualitative study. SAGE Open Med. 2013/01/01 ed. 2013;1:2050312113510001.

7. King LK, Krystia O, Waugh EJ, MacKay C, Stanaitis I, Stretton J, et al. Barriers and enablers to health care providers assessment and treatment of knee osteoarthritis in persons with type 2 diabetes mellitus: A qualitative study using the Theoretical Domains Framework. Osteoarthr Cartil Open. 2022/12/08 ed. 2022 Dec;4(4):100299.

8. Luijks H. Multimorbidity from the general practitioner’s perspective. Huisarts en Wetenschap. 2016;59(9):400–2.

9. Manias E, Claydon-Platt K, McColl GJ, Bucknall TK, Brand CA. Managing complex medication regimens: perspectives of consumers with osteoarthritis and healthcare professionals. Ann Pharmacother. 2007/04/26 ed. 2007 May;41(5):764–71.

10. Sav A, McMillan SS, Kelly F, Kendall E, Whitty JA, King MA, et al. Treatment burden among people with chronic illness: what are consumer health organizations saying? Chronic Illn. 2012/10/25 ed. 2013 Sep;9(3):220–32.

11. Sharp D, Lorenc A, Feder G, Little P, Hollinghurst S, Mercer S, et al. “Trying to put a square peg into a round hole”: a qualitative study of healthcare professionals’ views of integrating complementary medicine into primary care for musculoskeletal and mental health comorbidity. BMC Complement Altern Med. 2018/10/31 ed. 2018 Oct 29;18(1):290.

12. Sumpton D, Hannan E, Kelly A, Tunnicliffe D, Ming A, Hassett G, et al. Clinicians’ perspectives of shared care of psoriatic arthritis and psoriasis between rheumatology and dermatology: an interview study. Clin Rheumatol. 2020/09/17 ed. 2021 Apr;40(4):1369–80.

13. de Tommaso M, Sciruicchio V. Migraine and central sensitization: Clinical features, main comorbidities and therapeutic perspectives. Current Rheumatology Reviews. 2016;12(2):113–26.

14. de Rooij M, van der Leeden M, van der Esch M, Lems WF, Meesters JJL, Peter WF, et al. Evaluation of an educational course for primary care physiotherapists on comorbidity-adapted exercise therapy in knee osteoarthritis: an observational study. Musculoskeletal Care. 2020/01/28 ed. 2020 Jun;18(2):122–33.

15. Nawrot J, Boonen A, Peeters R, Starmans M, van Onna M. Rheumatologists’ Views and Experiences in Managing Rheumatoid Arthritis in Elderly Patients: A Qualitative Study. J Rheumatol. 2018/02/17 ed. 2018 May;45(5):590–4.

16. Diener HC, Gaul C, Holle D, Straube A, Storch P, Wallasch TM. Integrated headache care: Experiences from Germany, Denmark, and USA. Aktuelle Neurologie. 2012;39(9):467–74.

17. Edwards KA, Reed DE, Anderson D, Harding K, Turner AP, Soares B, et al. Opening the black box of psychological treatments for chronic pain: A clinical perspective for medical providers. PM and R. 2023;15(8):999–1011.

18. El Miedany Y. Comorbidity in rheumatic diseases [Internet]. 2017. 1–433 p. (Comorbidity in Rheumatic Diseases). Available from: https://www.scopus.com/inward/record.uri?eid=2-s2.0-85040238978&doi=10.1007%2f978-3-319-59963-2&partnerID=40&md5=0841b4eee6268afbfa93f0164298f569

19. Horton SC, Walsh CAE, Emery P. Established rheumatoid arthritis: Rationale for best practice - Physicians’ perspective of how to realise tight control in clinical practice. Best Practice and Research: Clinical Rheumatology. 2011;25(4):509–21.

20. Kvien TK, Balsa A, Betteridge N, Buch MH, Durez P, Favalli EG, et al. Considerations for improving quality of care of patients with rheumatoid arthritis and associated comorbidities. RMD open [Internet]. 2020;6(2). Available from: https://www.embase.com/search/results?subaction=viewrecord&id=L632398241&from=export http://dx.doi.org/10.1136/rmdopen-2020-001211

21. Brady N, Lewis J, McCreesh K, Dejaco B, McVeigh JG. Physiotherapist beliefs and perspectives on virtual reality-supported rehabilitation for the assessment and management of musculoskeletal shoulder pain: a focus group study protocol. HRB Open Res. 2022/02/16 ed. 2021;4:40.

22. Loza E, Lajas C, Andreu JL, Balsa A, González-Álvaro I, Illera O, et al. Consensus statement on a framework for the management of comorbidity and extra-articular manifestations in rheumatoid arthritis. Rheumatology International. 2015;35(3):445–58.

23. Prazeres F. Chronic pain and multimorbidity: Comment on "Chronic pain education in Portugal: Perspectives from medical students and interns”. Acta Medica Portuguesa. 2019;32(9):622.

24. Morrison D, Agur K, Mercer S, Eiras A, González-Montalvo JI, Gruffydd-Jones K. Managing multimorbidity in primary care in patients with chronic respiratory conditions. npj Primary Care Respiratory Medicine [Internet]. 2016;26. Available from: https://www.embase.com/search/results?subaction=viewrecord&id=L612205822&from=export http://dx.doi.org/10.1038/npjpcrm.2016.43

25. Radner H. How to improve care for patients with RA and comorbidities. Nature Reviews Rheumatology. 2020;16(11):607–8.

26. Ryan RE, Hill SJ. Improving the experiences and health of people with multimorbidity: exploratory research with policymakers and information providers on comorbid arthritis. Aust J Prim Health. 2013/03/21 ed. 2014;20(2):188–96.

27. Rijken PM, Dekker J. Clinical experience of rehabilitation therapists with chronic diseases: A quantitative approach. Clinical Rehabilitation. 1998;12(2):143–50.

28. Roberge P, Hudon C, Pavilanis A, Beaulieu MC, Benoit A, Brouillet H, et al. A qualitative study of perceived needs and factors associated with the quality of care for common mental disorders in patients with chronic diseases: the perspective of primary care clinicians and patients. BMC Fam Pract. 2016/09/14 ed. 2016 Sep 13;17(1):134.

29. Dinh TS, Brünn R, Schwarz C, Brueckle MS, Dieckelmann M, González AIG, et al. How do middle-aged patients and their healthcare providers manage multimorbidity? Results of a qualitative study. PLoS ONE [Internet]. 2023;18(8 August). Available from: https://www.embase.com/search/results?subaction=viewrecord&id=L2026850990&from=export http://dx.doi.org/10.1371/journal.pone.0291065

30. Schwarz T, Schmidt AE, Bobek J, Ladurner J. Barriers to accessing health care for people with chronic conditions: a qualitative interview study. BMC Health Serv Res. 2022/08/14 ed. 2022 Aug 14;22(1):1037.

31. Avgerinou C, Kotsani M, Gavana M, Andreou M, Papageorgiou DI, Roka V, et al. Perceptions, attitudes and training needs of primary healthcare professionals in identifying and managing frailty: a qualitative study. European Geriatric Medicine. 2021;12(2):321–32.

32. Balogun-Katung A, Carswell C, Brown JVE, Coventry P, Ajjan R, Alderson S, et al. Exploring the facilitators, barriers, and strategies for self-management in adults living with severe mental illness, with and without long-term conditions: A qualitative evidence synthesis. PLoS ONE [Internet]. 2021;16(10 October). Available from: https://www.embase.com/search/results?subaction=viewrecord&id=L2015309585&from=export http://dx.doi.org/10.1371/journal.pone.0258937

33. Bair MJ, Matthias MS, Nyland KA, Huffman MA, Stubbs DL, Kroenke K, et al. Barriers and Facilitators to Chronic Pain Self-Management: A Qualitative Study of Primary Care Patients with Comorbid Musculoskeletal Pain and Depression. Pain Med. 2009 Oct;10(7):1280–90.
