# Supplementary material for: Challenges and Perspectives in Treating Individuals With Musculoskeletal Disorders and Comorbidity: A Systematic Literature Review With a Descriptive Thematic Synthesis
Source: Scand J Caring Sci. 2025 Oct 3;39(4):e70130. doi: 10.1111/scs.70130 (PMC12495375; doi:10.1111/scs.70130)
Supplement: Supplementary file 6 — Data S6: scs70130‐sup‐0006‐Supinfo06.docx. [file SCS-39-0-s002.docx]

Appendix 6: All statements from included studies

**Study 1: Gibbs et al., [17]**

**Statement 1:**

The major barriers are really getting – the right nonsurgical treatment at the right time. . . Often, it’s socioeconomic, we really want to get into some non‐surgical management, but they just say they just can't afford it (OAHKS 2)

**Statement 2:**

There are plenty of situations where GPs are identifying that surgery probably isn't the answer and, particularly, where they need either expert opinion or they need multidisciplinary input, and that's very difficultto come by in primary healthcare. Equally, there are plenty of patients that come in saying “Look, I really don't want an operation. I'm here because my GP didn't know what else to do and so he sent me in. (OPSC 4)

**Statement 3**

So, the chronic disease management plans – you only get five sessions for allied health interventions, and so when you've got other comorbidities. . . five probably doesn't quite cut it for the year when a lot of people don't have the money to go privately. (OPSC 1)

**Statement 4**

Yeah, that system's [publicly funded non‐surgical care plans] definitely flawed.. . so many of the patients who get referred to it only go because there's no gap option (OPSC 4)

**Statement 5**

. . . the cost with the private GLA:D programme is definitely a barrier (OAHKS 1)

**Statement 6**

No barriers around financialbecause it's free. (OACCP 2)

**Statement 7**

We don't have a dietitian. . . so our only option is to do it privately and even with the GP management pro gramme the cost of a private dietitian is one of the barrier[s] to actually access a(sic) dietetics in the private sector (OACCP 3)

**Statement 8**

Some people, well quite a few who’ve been told it’s bone on bone and they’ve got quite significant pain

when they weight bear they’re quite pessimistic about prognosis if it’s not surgical, if its not the arthroplasty. (OAHKS 10)

**Statement 9**

GPs, a lot of them don't see the value in physiotherapy when it comes to hip and knee OA. They see this as a structural irreversible problem that is the cause of all the patient's symptoms and becoming more physically active or losing weight or following anti‐inflammatory diet, that none of those really have any meaningful value. And words that are often used by referring GPs is, “I am seeking a definitive solution. Therefore, hip arthroplasty is indicated.” (OPSC 5)

**Statement 10**

It is challenging because GPs are telling them, “Oh yeah, I'm referring you to the hospital for a knee replacement” (OPSC 3)

**Statement 11**

I think the other thing that makes it really hard is use [of] the term ‘bone on bone’. So probably 90% of people. . . from the surgeons which have [been told it's] bone‐on‐bone disease, their joints are crumbling. . . I think it's really hard when a doctor's used that emotive language to say, “Oh, you know the x‐rays tell a story, pain doesn't equal damage” (OACCP 3)

**Statement 12**

. . . essentially, they come in for orthopaedic service. Often, the patient will still want the orthopaedic surgeon to give them that same opinion, like they don't quite trust your opinion. (OAHKS 8)

**Statement 13**

If they [patients] have been told it's the end‐stage arthritis bone on bone, those sorts of words can really stick with the patient and sometimes, they come into the OAHKS clinic thinking that they just need the operation and that's the only solution to their problem (OAHKS 4)

**Statement 14**

OAHKS in the community setting might be a better way to run it, better than getting the patient to come into an acute hospital setting if there's a similar type of setup in a community health centre. Maybe that might change patient expectations to some degree (OAHKS 4)

**Statement 15**

Those [patient's who value doctor over physio] are the clients that'd be having that good liaison with the ortho guys here, if they're around, will actually pop in and explain that to a patient (OPSC 3)

**Statement 16**

If you’re seeing someone with a BMI of 50 and they’ve got severe knee OA, and they’re waiting for surgery, and they’ve got mental health issues because of childhood trauma. . . that’s a tricky patient to help lose weight (OPSC 1)

**Statement 17**

Weight management is another issue altogether. That’s a bit tough to get effective management for that. We don’t have any real answers. (OAHKS 9)

**Study 2: Hemmings & Soundy, [18]**

**Statement 1**

‘I feel people are not listening to me’ (Patient one)

**Statement 2**

‘the person doesn’t seem to have listened to what’s going on- the pain but the context of you know the pain exists but there are other things that are going on which may contribute to the pain’ (Patient 3 )

**Statement 3**

‘when I’ve gone to see an NHS physio – and they’re probably overworked whatever- they don’t really ask the questions about what’s really the matter and they suggest things that you just think ‘oh it’s pointless’’ (Patient 8)

**Statement 4**

I hope that they’d explain everything- not just give me a handout of exercises…‘it don’t make sense to me and I can’t remember exactly how to do it properly (Patient 4)

**Statement 5**

‘if they give me a task to do, check that I’m doing it right ‘cause I don’t follow written instructions very well’ (Patient 5)

**Statement 6**

‘I like a treatment plan- I like to be kept updated that’s what sometimes I feel like I’m getting lost with what’s going on’ (Patient 5)

**Statement 7**

‘they assume they just have to fix your body and someone else is there to fix your brain’ (Patient 2)

**Statement 8**

‘to you as a physiotherapist you’re only looking at one problem but to me as a patient I’ve got multiple problems. I’m trying to juggle things and sometimes I can’t physically juggle everything on top of what’s going on with me’(Patient 3)

**Statement 9**

How can like anyone- a friend understand- and when you get medical staff who don’t understand? (Patient 4)

**Statement 10**

It’s the understanding their problems that they don’t necessarily get from other people [physiotherapists] sometimes (Physiotherapist 2)

**Statement 11**

‘because of the depression and it was just too much to do’ (Patient 4)

**Statement 12**

‘It wasn’t beneficial and I just lost heart’ (Patient 4)

**Statement 13**

‘you don’t feel the benefits of the session because you’re actually too stressed out’ (Patient 8)

**Statement 14**

Whenever I work out or do exercise my mind gets clearer and I feel a lot better mentally (Patient 1)

**Statement 15**

felt mentally better ‘cause I felt like I was taking care of myself’ (Patient 5)

**Statement 16**

‘due to the situation of being in a mental health hospital, restrictions and time consumptions and other like quantity of seeing patients - making sure it goes round - it was lacking. I would say there weren’t enough physiotherapists to go around’ (Patient 1)

**Statement 17**

‘other times the GP has referred me for physiotherapy and nothing has happened’ (Patient 8)

**Statement 18**

I just got lost from the system (Patient 5)

**Statement 19**

I have physio at my GPs… like a conveyor belt… you’ve gotta get here for this time… we’re not seeing you if you don’t attend… you’ve gotta go through the process again…

**Statement 20**

it’s quite a lengthy process… the NHS is slow now and you have to go through a lengthy timescale (Patient 3)

**Statement 21**

‘I love 1-1 because it really helps…I can be motivated but I like being accompanied’ (Patient 1)

**Statement 22**

‘to actually get yourself out of the house can be quite stressful’ (Patient 3)

**Statement 23**

‘I’ve always gone when they [give an appointment]’ (Patient 6)

**Statement 24**

‘if I’ve got an appointment, I’d move heaven and earth to be there’ (Patient 8)

**Statement 25**

Struggled to attend physiotherapy ‘due to mental capacity issues and lack of motivation’ Patient 1)

**Statement 26**

‘you feel like you can’t be bothered with this because it’s another thing that adds onto the rest of it’ (Patient 3)

**Statement 27**

If a person can’t be bothered to do it, they won’t do it (Patient 2)

**Study 3: King et al., [19]**

**Statement 1**

“Yeah, because it’s [osteoarthritis is] not as simple as someone coming in and they have a rotator cuff strain… I think the patients are a bit more complex in terms of their comorbidities, and almost all of them have several chronic health issues.” (ID 11)

**Statement 2**

The n, if we’re looking at somebody [with diabetes] that’s inactive currently, I advise them to speak to their family physician before they start any new activity-based program. That’s to kind of cover my butt, because I’m not comfortable with the management of it. It’s just sort of like, I don’t want to throw everything out of whack in your sugars if you’re used to not doing any activity. So, just check. (ID 05)

**Statement 3**

I find that people that are overweight have other issues. I can’t go open that can of worms because I don’t know what to do with it. (ID 17)

**Statement 4**

Not really, because I’ve never done them [behavior change techniques], so I would say not familiar. Whether I learned them at some point in my undergrad 15 years ago, but I don’t use them, so I would say I’m not familiar with them (ID 11)

**Statement 5**

Yeah, we have definitely done some stuff around that. I definitely try to do some of that. To be perfectly honest, there are probably people in the group that maybe are better at it than me, but I do make an effort. (ID 01)

**Statement 6**

I guess it goes hand in hand [OA and diabetes]. We’re dealing with people with age-related changes, OA, they may be overweight. Yeah, I do think we have a role. We talk about weight loss. I certainly bring in healthy eating too and refer them on to dieticians if necessary. Yeah, I do think we have a role, but I guess our focus is primarily the arthritis, right, whereas the doctors are focusing on the diabetes. (ID 14)

**Statement 7**

I don’t think it [diabetes] maybe majorly changes the way I would recommend activity. Of course, it would be in consideration, but in terms of my knee OA management, I must say I don’t think it would make a huge difference in terms of recommendations. (ID 07)

**Statement 8**

I think I absolutely take that [comorbidities] into consideration because there’s a lot of various conditions that people with diabetes will have, that can get mixed up in arthritis. (ID 12)

**Statement 9**

But probably on average I would see a normal OA case twice. (ID 13)

**Statement 10**

I think it just gives you more credibility and makes them more accountable if they know that someone is checking in on them. (ID 18)

**Statement 10**

“If I understood that a bit better, then I might be able to communicate that [importance of weight management] to them better. That would be useful, and I guess I would probably need some information on whether or not I’m supposed to be modifying the physical activity, depending on whether or not they have diabetes, because I don’t think I really have up until now.” (ID 01)

**Statement 11**

“So what’s challenging for me is I don’t want to say or do the wrong thing….” (ID 18)

**Statement 12**

“I would definitely feel more comfortable having more education around how diabetes in general is managed and the different medications and that sort of thing, as well as the impacts of exercise on that.” (ID 05)

**Statement 13**

“It’s probably more something that I’ve heard of as opposed to used.” (ID 05)

**Statement 14**

“I do have my Bachelor of Education, so I think that probably helps in terms of creating goals, etc. In physio school, it’s somewhat talked about, for sure, but I think a lot of that, for me, has come more from my BEd.” (ID 07)

**Statement 15**

“I actually use health coaching, I’ve spent a fair bit of time in my latter career, really, learning that cool technique of health coaching. I ask them, I try and get them to identify what they think they should do, and get it down to something that’s manageable, that they feel they can succeed at, if that makes sense.” (ID 12)

**Statement 16**

“I was just talking with my director recently about how I want to take a course on this [behavioral change strategies]. Because I think it’s so important, and, you know, it’s an important skill in working with these patients. So, I don’t feel mine is great and I definitely want to work towards that.” (ID 03)

**Statement 17**

“I would say probably I would … especially in this particular job, I don’t think that it would be my role [to optimize concomitant diabetes in patients with OA].” (ID 12)

**Statement 18**

“…You have to remember as a physio myself my strength is biomechanics and how people move.” (ID 02)

**Statement 19**

“So we talk about it [comorbidities such as diabetes] a little bit, we don’t, you know, that’s not my area of expertise so I don’t go into great detail, but that is something that we talk about.” (ID 03)

**Statement 20**

“I think I currently just ignore that piece [comorbidity]. If they tell me they’ve got diabetes, I just write it in their medical history part and I don’t address it, or have it influence any of my recommendations, really. So, if I were to need anything, probably it would be education, if there are ways that knowing they have diabetes should be influencing my recommendations, because I just wouldn’t even know.” (ID 13)

**Statement 21**

“I think, yes, in the sense that I can tell them that exercise would help manage their diabetes, and a lot of the things that go with that, so let’s say their high cholesterol or something like that. Yes, from an education piece, yes.” (ID 11)

**Statement 22**

“If it’s [diabetes] really not under control, then I would probably try to spend a little bit of time explaining how blood sugar, the insulin response and the effect of sugar on the body, is known to cause some of that increased inflammation and joint pain, so trying to make them understand the link in-between joint pain and their diabetes.” (ID 01)

**Statement 23**

“Or when it’s coming to exercises or increasing exercise, I say, what do you think is a realistic starting point for completing these exercises, what can you see yourself realistically doing, to try to get them more involved. And so, we set something specific, and then I remind them that at our follow-up call I will be asking about it. But I don’t think we’re well-suited for long-term accountability. It works for our first follow-up, but I’m not going to book another follow-up just for accountability.” (ID 13)

**Statement 24**

“I’m not sure [that current model of education and self-management is sufficient] because we don’t [follow-up]… because our mandate is to educate, self-management. We don’t follow them after that. I don’t really know what happens with that. I hope and think that it does kind of contribute to a lot of management, but I’m not sure.” (ID 17)

**Statement 25**

“I am probably a little more cautious because of possible cardiovascular risk factors. Because I’m not in the position to measure or observe some of the risk factors that they might be able to be monitored on if they were at a cardiac rehab prevention program, like those programs where they’re taught.” (ID 06)

**Study 4: Lawford et al., [20]**

**Statement 1**

Alex: often when people with OA come in they’ll use terminology like “Oh, no I’m bone-on-bone” and things like that and they just, any sort of impact is going to worsen their symptoms. So I suppose providing some education to people, that strengthening exercises, weight bearing exercises is not necessarily going to make it any worse and in a lot of cases, will make it better.

**Statement 2**

William: a lot of people were highly sceptical. Highly sceptical. And that was a big flag with a few - some people came in thinking this is a crock of [rubbish], this is not going to do anything, blah, blah, blah, and they were hard to work with. To get that buy in and that rapport, it was quite a challenge in those first couple of sessions

**Statement 3**

William: I think fear is quite a big thing in that group, particularly because we’re asking them to do functional things like stairs and people are quite scared of pain…I think that the study forced me to push people through more pain than, perhaps, I would have previously, and I’ve learnt positively from that

**Statement 4**

Mary: a lot of the patients I had would come in, and they were very fixated on their pain and the effects of the pain on their lifestyle…A lot of them just couldn’t get past the pain in the knee…a barrier would definitely be beliefs and attitudes about pain and how much a patient would be prepared to push through a bit of pain.

**Statement 5**

William: these are also people that don’t particularly like exercise. It hasn’t been important to them and they’re fatigued, and they’ve got a low work capacity. They’re not particularly fit and exercise is something they don’t always view positively.

**Statement 6**

Mary: The [WBE] group would find those exercises more of a mental - mentally tiring. Focusing, concentrating, than actually getting an actual muscular exertion sense…for them it was not about the load on their muscles necessarily, it was about how much cognitive effort it was. Mental effort, for them to do the right alignment

**Statement 7**

Aiden: the NWBE protocol was a lot easier for [patients] to follow [than the WBE]...just because it was less technical

**Statement 8**

Alex: [in private practice] we can tend to fall into a bit of a trap of maybe not pushing people…how much some of the participants could do probably surprised me - with both of the treatment protocols [we] were able to do some pretty tricky exercises and there wasn’t an increase in their symptoms…initially I was anticipating that a lot of these people - being overweight and OA changes, they may not be able to handle a huge amount of exercise. But, as I said, I was pleasantly surprised with that.

**Statement 9**

Aiden: It was interesting to see that you could challenge particularly with the weighted work, that most of the knees tolerated a lot heavier loads that probably clinically in the past I probably would’ve put on people, in the physiotherapy setting

**Statement 10**

Aiden: the [WBE] protocol has a little bit more finesse and does require a person to have a little bit more, I don’t know, body awareness to get it right...the NWBE one was easier to do just because it was less technical

**Statement 11**

Neil: it’s obviously really specific about technique with those [WBE] exercises…especially obviously these people are overweight so they haven’t done a great deal of exercise or anything along those lines prior to this…[NWBE] was a lot easier to get because it’s less reliant on technique, so it was a lot easier to get the patients to actually have an understanding of what they needed to.

**Statement 12**

Mary: The cuff weights were awkward - Difficult to put on for patients, and me, at times. I found them awkward myself. Quite often, their abdominal bulk – because these are all bigger people – that their abdominal size meant it was hard for them to get down and put those ankle weights on

**Statement 13**

William: the cuffs were really cumbersome…they’re highly uncomfortable and really bite into their skin - and a lot of participants had trouble putting them on themselves due to poor mobility. They didn’t have the hip and spinal flexion to be able to get down to put the cuffs on - And they couldn’t climb on the floor because they couldn’t get back up again

**Statement 14**

Bob: [the difficulty] would 100% be the straight leg raise… That was the one that I found had a lot of issues, whether it was flaring up hip or groin pain, or low back pain. It was also the one that was probably the most challenging to progress throughout the five sessions.

**Statement 15**

Mary: the straight-leg raise was an exercise that some patients did that with no weight the whole way through, because it’s very long lever, long loaded exercise. So, for quite a lot of patients, we didn’t add any weight at all, just because they would get too sore at the front of their hip or cause back pain…some patients we just didn’t do it at all, because they hated it, and they were going to stop the study because of that one exercise.

**Statement 16**

Alex: There were a couple of people who withdrew from the study just due to other aspects of their life. They had things going bit pear shaped in their personal life. Another guy got like cancer and there were some understandable reasons why people withdrew

**Statement 17**

Simon: I had a couple of people pull out for medical reasons - one cancer, and one's partner died, and that sort of thing. … there was a few medical things where they sort of had to drop things and attend to other things

**Statement 18**

Mary: one lady’s mum had a stroke, so she went up to Queensland and she had to stay up there…another lady got sick, and she kept getting sick, so she couldn’t get in.

**Statement 19**

William: To get that buy in and that rapport, it was quite a challenge in those first couple of sessions. But they actually started seeing improvement and their attitude changed and they were easy to work with after that.

**Statement 20**

William: I think we had good rapport. I think I got on quite well. I certainly got some good feedback from a couple of patients…It’s not just a clinical interaction. You’re treating a person. You’re not treating a problem. And I think that helps with compliance too

**Statement 21**

Mary: I had a really nice rapport with the vast majority. Yes. There’s a couple that probably - I had to work a bit harder with a couple of them to develop rapport, because they came in with – well I suppose negative attitudes – to whether this was going to help them…You have to work hard to win people over sometimes. Harder with some that are very set in their beliefs.

**Statement 22**

Bob: definitely some form of pain education or lots of reassurance. It was a bit tricky for people to exercise comfortably or out of apprehension - I think a lot of people were quite reluctant to go further into that exertion level. So you would need some form of guidance, reassurance

**Statement 23**

William: If they could see why they were doing it, I think that helped the buy in as well…I said these exercises are purely trying to make you put a bit of weight through that leg and educating that it’s okay and it’s safe to do so…they definitely need to be encouraged and feel like that they can achieve the exercise.

**Statement 24**

Simon: I think once they got their head around the idea that if they push themselves it's going to help them and they expected a bit of pain - then they were fine. I found the people that were quite pain avoiders, they were the hard ones to get going. But that's just understanding those concepts of what pain is and that sort of stuff.

**Statement 25**

Mary: the stiff knees tended to cope better and do better and feel better with the [NWBE group]. And the looser knees, more mobile knees, tended to do better with the [WBE] exercises…You’ve just got to find what’s going to work for the patient

**Statement 26**

Aiden: overall, I think, if you have the right patients, the WBE one is fine and the [NWBE] one might be the person learning the exercises and there isn’t that heavy grinding or clunking feeling when they loaded.

**Study 5: Teo et al., [21]**

**Statement 1**

Beatrice: ‘Being more thorough with checking your red ﬂags and looking at what’s happening above and below the joint. Looking at the knee itself, ruling out any actual pathologies like ligament injuries or maybe it might be gout instead of OA, and you have to just take a good picture get the aggravating and easing factors and go from there.’

**Statement 2**

Mary: ‘I want to know where the pain is, whether it’s conﬁned to the knee or whether they’re having problems in other areas. look at their range of motion, have a feel around the knee. Sometimes they’ve got stiff patella or stiff tibiofemoral joints. Sometimes their muscles around that area are tight or tender.’

**Statement 3**

Eric: ‘I generally just take a history of the person, their age, onset of symptoms, where their pain is in their knee, how long they’ve had pain in their knee, and to rule out other things that might be going on in the knee.’

**Statement 4**

Jean: ‘I guess load management is the big thing in that regard – what they’re doing at home, and activities of daily living. If it’s a housewife that likes to clean the house every day, giving instruction in regards to that: you shouldn’t be doing any deep squatting, no kneeling. Trying to avoid anything that’s going to load bear on the knee.’

**Statement 5**

Mary: ‘I tend to want people to understand about pacing, so pacing being fairly consistent from day to day how much activity you do so you don’t have one day where you garden for ﬁve hours and then the next day where you sit on the couch for ﬁve hours – trying to think about breaking activity down over the course of a week so that it’s fairly steady, and doing a little bit of activity and then changing activity, preferably not just resting.’

**Statement 6**

Janice: ‘If they have a reduction in knee ﬂexion, I’ve found that there’s some mobilisation techniques that you might use just to sort of increase ﬂexion or extension movements there. Even if it’s just to sort of get an immediate short-term pain beneﬁt I think sometimes patella mobilisations or quads, soft tissue massage.’

**Statement 7**

Janet: ‘Osteoarthritis being a wear and tear problem with the joint and that a lot of the pain comes from the inﬂammation that goes along with that.’

**Statement 8**

Steve: ‘This is partly due to the wear and tear, so it’s aging like your hair, like your nails; your joints do age.’

**Statement 9**

Sandy: ‘When I start to explain OA, is about “Over time, basically the joints start to wear down, usually from loading.” It can be other factors but most of the time is from loading.’

**Statement 10**

Janice: ‘Of course function, so what they can do, what they can’t do and I guess what they’d like to be able to do. Because I guess that then gives you and them something to work for with regards to goal setting.’

**Statement 11**

Mary: ‘I usually dig around quite a bit to make sure I get a good picture of their normal activity levels and how that has changed from prior. And then I guess I want to know whether their goals are to get back to those or whether they have something else.’

**Statement 12**

Gerry: ‘Just about trying to work with what their needs are and what their goals are.’

**Statement 13**

Janice: ‘Functionality if they can sit to stand, if they can squat, if they walk up and down stairs, what their range of motion is like. I guess – yeah you want to try to as much as possible to tailor it to increasing their function, basically.’

**Statement 14**

Nicholas: ‘How far can they walk because that’s the main limitation that most people with medium to high-grade knee osteoarthritis seem to have. So, if we can get a good measure of that then we can monitor that, reassess that, and reassess their improvements over time.’

**Statement 15**

Ryan: ‘Most important assessment in my opinion would be functional outcomes. So, depending on what they can do, because most of the time the OA knee patients, they come in, they come with a lot of pain, and you can’t ﬁx all of them, but then the most important thing is you want them to be able to achieve their own goal of being able to walk a little bit better, go out shopping.’

**Statement 16**

Alice: ‘Ask them what they are interested in – there is no point in telling someone to go to a pool and walk if they hate the water, hate bike riding, then they are not going to do it – ﬁnd out what they are interested in, what worked for them in the past. Do they like group exercises or not? Are you better off if we create a program for you to do it at home?’

**Statement 17**

Jean: ‘So to start with, the non-weight bearing, so the exercise in the hydrotherapy pool, also – either lying or sitting VMO work – some active range of movement can be quite nice – as well if they’ve got some hamstring or calf tightness. When their symptoms start to decrease, progress to weight bearing whether it be terminal knee extension against the wall, pressing back with a ball, terminal knee extension with a resistance band – then into functional movements because that’s what they’re going to have to return to.’

**Statement 18**

Steve: ‘If weight-loss is a contributing factor then you’re talking about referral to a dietician – plus talk to them about non-weight-bearing cardio exercises. I like cycling if the joint is happy for that bending and extension if that’s not irritable. I’d probably start with cycling because it’s less weight-bearing and then work on slowly progressing that.’

**Statement 19**

Ryan: ‘I’d typically get them in say twice a week for the ﬁrst one or two weeks, depending on how bad they are, then I’d probably spread them out to once a week, or even like once a fortnight, if they are progressing well. When they’ve reached their goal, say pain free, get back to sports, or get back to whatever it is they used to be doing, then it’s time to discharge.’

**Statement 20**

George: ‘If they need a bit of reassuring – I might see them a bit sooner. The ones that are more self-sufﬁcient and able to manage things by themselves, I might review weekly or fortnightly or every couple of weeks. Once they’ve got a good programme going, maybe monthly. There’s some that have no interest in doing any exercise classes but they’re happy to manage things by themselves at home. They might be the one I see at ﬁrst maybe every two weeks and then monthly after that. And then once they are happy with their symptoms and how things are progressing, I won’t continue seeing them on a regular basis and I might just touch base via phone call.’

**Statement 21**

Jean: ‘Ok, day one. If I’ve given them something and I’m not sure whether they’re going to cope with it or what the effect may be, I might see them again in seven days’ time. And then if they’re going well, I might give them another one or two things, and they’ll go away and work on that for another three weeks or so. I usually review on a two to three weekly basis, and then if I’m happy that they’re managing their programme, I might stem it out to a month review – when they’ve gotten back to their baseline, I’d be discharging.’

**Statement 22**

Janice: ‘They have a general practitioner or an orthopaedic specialist already who has booked them in for surgery or has decided that that’s the path ahead. I’d still encourage them always to exercise even if obviously, they’re going to have surgery, they’re going to be pretty painful. They can try to get a little bit of weight loss before surgery knowing that they will beneﬁt more and get more out of.’

**Statement 23**

Mary: ‘If I’m seeing someone who is already booked in for surgery, then I’ll talk through the expectations of that surgery and the postop period. If someone’s not booked in, I usually don’t even raise the topic.’

**Statement 24**

Jeremy: ‘When clients come in with knee OA, often they’re on the waiting list already for having a knee replacement. Not many of them would understand what the actual surgery is. We have pamphlets that actually explain what a knee replacement surgery is, and I’ll go through that with them and expected timeframes for recovery.’

**Statement 25**

Ryan: ‘A lot of the time they have a lot of comorbidities, like I’ve got one who came in with ﬁbromyalgia, knee OA, RA, and plus complex regional pain syndrome. These will all factor into pain. And yeah, it makes it a lot, lot harder to manage.’

**Statement 26**

Gerri: ‘Probably the two main challenges would be co-existing comorbidities, so often they (are) overweight, or they also have cardiovascular disease. They may also have high cholesterol, high blood pressure.’

**Statement 27**

Sharon: ‘I’d imagine if I had obese people who weren’t able to lose the weight that would be challenging. And I’ve had a few where it’s just so advanced that they just can’t do anything and especially like the very elderly where they just can’t have surgery and they also have like so many comorbidities in regards to pain. That’s challenging.’

**Statement 28**

Nicholas: ‘The main challenge we ﬁnd is the actual compliance – the patients doing their exercises – How do we get this going on a long- term basis? How can we maintain? Yes, they’ve come and seen us in the last three months and improved a lot, but we can’t keep seeing them for the next ﬁve, six, 10 years.’

**Statement 29**

Gerri: ‘Well, one challenge here, where I live, is that it’s very cold, and nobody likes to go outside for a walk. If they have had a fall, they can be very nervous about exercising alone, even to go for a walk, so there’s that fear of falling. Fear of falling, the cold weather, they would be the key challenges.’

**Statement 30**

Alice: ‘I try and get them to have a routine, to visualise what time and day they will exercise, days they will do it, and what room they will do it in, so that they get a pattern. What type of equipment they will use, help them work that out so that they have got a deﬁnite plan rather than send them home and they are left with their own devices, so they are less likely to do it.’

**Statement 31**

Ben: ‘Some people will just want a quick ﬁx and if your message to them is that they have to manage their life, that they may have to lose weight, that they may have to exercise – people don’t really take to that. They want the surgery; they want the pill. They want someone else to ﬁx them.’

**Statement 32**

Beatrice: ‘They still just want the passive quick-ﬁx treatment. They want me to chuck a machine on them. They want me to rub their leg and tell them everything’s okay. And they don’t want to own their condition.’

**Statement 33**

Andy: ‘That they have a preconceived expectation that surgery is the only option. They’re not willing to change their opinion on the treatment that they need.’

**Statement 34**

Nathan: ‘We never use imaging as a prime focus; we just look at the other scenarios such as range of motion limitations, effusion, swelling in their knees, their background, whether they have had a traumatic background or whether it’s a gradual onset. Then if I have the capacity and the time and the ability to do it, then I’ll normally order an x-ray just to double-conﬁrm just to see if the imaging kind of guides what came out from the subjective assessment.’

**Statement 35**

Sandy: ‘I do ﬁnd that even if I said what I think is going on in the knees are OA and explain it, a lot of the clients still go off to the doctor to get an imaging. I usually end up getting that sort of imaging once I have made the diagnosis as a support. [For example] ‘joint line space degeneration in the knees’ In that regard, the diagnosis that I have given in regard to OA in the knees has been right because they went and got imaging afterwards.’

**Statement 36**

Jean: ‘Often it’s hard to convince them of the diagnosis without imaging, even though imaging isn’t warranted necessarily in this patient group – they want that imaging done to conﬁrm. And they’re very hesitant to take your word for it that there’s osteoarthritis there. They want that proof, they want to see visually, internally, that yes this is wear.’

**Statement 37**

Sandra: ‘I do explain to them that the extra weight is putting extra force through their knee and so causing extra pain – I also ask them just vaguely – I touch on what they’re actually eating throughout the day. And most people say ‘oh I have a couple of bikkies at morning tea and then an ice cream for dessert’ or something.’ I just generally brush on ‘Don’t try and cut it all out but what if we just cut back to one bikkie instead of two and maybe half that bowl of ice cream, or every second day?’

**Statement 38**

Janet: ‘If patients report they are at a healthy weight, we talk about how important it is to maintain that healthy weight and getting diet and exercise to the right levels to do that. for patients whose BMI wouldbe in the overweight range,wewouldhelp with their weight management at a physiotherapy level. We work out what activity they can do that doesn’t increase their pain and symptoms, the duration and the intensity at which they can do that. We give them education about basically calories in versus calories out approach to weight loss.’

**Statement 39**

Ryan: ‘I will mention to them, it was each kilogram you lost and your weight will reduce 10%. usually they’re quite sedentary, so start with some basic exercises like walking, or even a bit of cardio-type exercise or simple exercise at home, and give a little bit of advice in terms of diet – like drink plenty of water and a balance of green vegetables and meat.’

**Statement 40**

Michael: ‘There’s not a lot of evidence long-term for manual therapy but I think it provides a small window that clients then feel a bit more at ease to move and more willing to exercise.’

**Statement 41**

Mary: ‘I do occasionally use manual therapy even though I know that’s not evidenced based. But for the right client sometimes a bit of mobilising, patellofemoral or tibiofemoral joint or some soft tissue work – often the lateral distal quads are quite tight and uncomfortable and a bit of work around there can take some pressure off the knee. I’m very aware that that’s not evidenced based, but I ﬁnd in practice that it does work. I do that occasionally when it seems relevant.’

**Statement 42**

Sharon: ‘Many physios would absolutely kill me for this – but I still think that manual therapy with OA in the beginning is under-utilised. I think it’s becoming a thing now to just started loading them – they respond so well to lots of good release work around their hip and knee and I just think that that whole element is being just sucked out of a physio; ‘They don’t need to be touched.’ – I kind of disagree with that.’

**Statement 43**

Sandy: ‘I do talk about the importance of weight, how the more you weigh, the more shock, and the more absorption it needs to be doing, but I don’t push it too much, because I think that there are other health professionals who have a better way of approaching it compared to me.’

**Statement 44**

Beatrice: ‘I mean, I’m not a weight loss expert. I’m not a dietician. I’ll often say to patients if they’ve got a high body mass index, weight is very much a contributing factor to their knee. Some simple things they can do is think about diet and exercise and go and have a chat to their general practitioner. If not, we can link them in with a dietician or nutritionist – if they need further help.’

**Statement 45**

Janice: ‘If their pain is not being managed well then generally I’ll refer them back to the doctor to discuss if they need something stronger. We know that stronger opioid medications are probably not going to be of much use to them – but I don’t generally give advice on what to take and what not to take. If we discuss it, I generally just advise them to run whatever they’re thinking by a general practitioner or a pharmacist.’

**Statement 46**

Jeremy: ‘If they’re struggling to bend the knee or ﬁnd that everything’s too irritable for them to start exercise, I might consider talking to their doctor. But I normally leave that to the doctors, I must say, rather than me giving advice with medicine because I feel that’s a bit outside my scope of practice.’

**Statement 47**

Nathan: ‘We then refer them on to see the surgeons anyway – we leave the surgical advice to them and they’ve got all the necessary documentation for the patient to take home in terms of information sheets, et cetera. it’s all sort of managed through them. But we generally tend to not step on that boundary if we can. We could but we don’t.’
